# Targeting colorectal cancer with pyrazolo[4,3-*e*][1,2,4]triazine and pyrazolo[4,3-*e*]tetrazolo[1,5-*b*][1,2,4]triazine sulfonamides: comprehensive in vitro evaluation

**DOI:** 10.1038/s41598-026-52451-5

**Published:** 2026-05-31

**Authors:** Mateusz Kciuk, Gabriela Machura, Katarzyna Wanke, Sylwia Smarzewska, Mariusz Mojzych, Elżbieta Płuciennik, Renata Kontek

**Affiliations:** 1 Department of Molecular Biotechnology and Genetics, University of Lodz, Banacha 12/16, 90-237 Lodz, Poland; 2 Faculty of Medicine, Department of Functional Genomics, Medical University of Lodz, Zeligowskiego 7/9, 90-752 Lodz, Poland; 3 BioMedChem Doctoral School of the University of Lodz and Lodz Institutes of the Polish Academy of Sciences, Lodz, Poland; 4 Department of Inorganic and Analytical Chemistry, University of Lodz, 12 Tamka Str, 91-403 Lodz, Poland; 5 Doctoral School of Exact and Natural Sciences, University of Lodz, Banacha Street 12/16, 90-237 Lodz, Poland; 6 The Mazovian Academy in Płock, Collegium Medicum, Plac Dąbrowskiego 2, 09-402 Płock, Poland

**Keywords:** Cancer, Drug, Pyrazole, Tetrazole, Triazine, Biochemistry, Cancer, Chemical biology, Chemistry, Drug discovery

## Abstract

Colorectal cancer (CRC) remains a major global health challenge, ranking as the third most common malignancy and the second leading cause of cancer-related mortality worldwide. This study aimed to evaluate a novel series of pyrazolo[4,3-*e*][1,2,4]triazine and pyrazolo[4,3-*e*]tetrazolo[1,5-*b*][1,2,4]triazine sulfonamides as potential anticancer agents, with a focus on their cytotoxic and mechanistic effects against CRC cell lines. Incorporation of the tetrazole ring significantly increased cytotoxic activity (up to 50-fold) and selectivity (3.6-9.2-fold) compared to non-tetrazole analogues (2.2–3.3). Pyrazolo-tetrazolo-triazine derivatives exhibited IC₅₀ values ranging from 0.12 to 1.1 µM and demonstrated enhanced solubility and consistent biological activity. Selected derivatives (MM134, MM136, MM137, MM139) induced marked DNA damage and inhibition of cell proliferation, with cell cycle analysis confirming apoptotic and necrotic features depending on the compound and exposure time. Electrochemical analyses confirmed direct interactions between MM compounds and double-stranded DNA. Notably, MM137 was the potent inducer of cell death in both HCT-116 and HT-29 3D spheroids at concentrations as low as 5 µM, comparable to the most pre-clinically advanced MM129 derivative. In conclusion, the study highlights pyrazolo[4,3-*e*]tetrazolo[1,5-*b*][1,2,4]triazine sulfonamides as promising anticancer candidates with potent cytotoxicity, favorable selectivity, and potential multimodal mechanisms of action.

**Supplementary Information:**

The online version contains supplementary material available at 10.1038/s41598-026-52451-5.

## Introduction

Colorectal cancer (CRC) stands as a formidable global health concern, characterized by its substantial incidence and mortality rates. As the third most commonly diagnosed cancer worldwide, CRC presents a significant burden to healthcare systems and populations across diverse geographical regions^1^. The etiology of CRC is multifactorial, encompassing dietary habits, lifestyle choices, genetic predispositions, and age. The prevalence of CRC escalates with age, predominantly affecting individuals over the age of 50^1–4^.

Despite advancements in screening and treatment, CRC remains the second leading cause of cancer-related deaths globally. Mortality rates exhibit a downward trend in developed countries due to enhanced screening initiatives and newer therapeutic interventions. Survival outcomes are closely linked to the stage at diagnosis, with early-stage CRC demonstrating significantly higher survival rates compared to advanced stages^1,5^.

Chemotherapy serves as a pivotal component in the management of CRC, either as adjuvant therapy to eliminate micrometastatic disease post-surgery or as neoadjuvant therapy to reduce tumor burden preoperatively. Despite these advancements, the prognosis for advanced CRC remains poor, highlighting the necessity for continued research and clinical trials to explore novel therapeutic avenues^6–9^. The exploration of heterocyclic compounds has provided a rich avenue for drug discovery, given their diverse biological activities and structural versatility^10,11^.

Compounds that contain a 1,2,4-triazine nucleus that is condensed with five-membered heterocycles have garnered a significant amount of attention because these compounds are bioisosteric with a purine core. Compounds containing a pyrrole ring, such as pyrrolo[2,1-*c*][1,2,4] triazine and pyrrolo[2,1-*f*][1,2,4] triazine, represent the most abundant class of triazine derivatives with antitumor activity. Mojzych et al. generated a series of pyrazolo[4,3-*e*][1,2,4] triazine derivatives and tested their cytotoxic activity in various cell lines^12^. Pyrazolo[4,3-*e*][1,2,4] triazines exhibited inhibitory activity towards histone deacetylases (HDACs)^13^, metalloproteinases^14^, tubulin^15^, urease and tyrosinase^16,17^. The anticancer activity of pyrazolo[4,3-*e*][1,2,4] triazines was also reported to significantly improve with the addition of a triazole or tetrazole ring to the compound structure^12^. To fully enhance the biological activity of pyrazolo[4,3-*e*][1,2,4] triazines and pyrazolo[4,3-*e*]tetrazolo[1,5-*b*][1,2,4] triazines, these heterocyclic cores were modified to harbor a sulfonamide moiety – presence of a sulfonamide group (–SO_2_NH_2_) attached to an aromatic or aliphatic functional group. The sulfonamide moiety is typically utilized in medicinal chemistry as an efficient bioisostere of the carboxylic group and has the potential to avoid some of the disadvantages that are associated with the incorporation of the former into heterocycle structure, including metabolic instability, toxicity, and limited passive diffusion across biological membranes. The FDA has approved the use of several sulfonamide-bearing compounds for the treatment of cancer. These include belinostat (HDAC inhibitor), ABT-199 (B-cell lymphoma 2 (BCL-2) protein inhibitor), or amsacrine (topoisomerase II inhibitor)^18^.

The anticancer activity of pyrazolo[4,3-*e*][1,2,4] triazine and pyrazolo[4,3-*e*]tetrazolo[1,5-*b*][1,2,4] triazine sulfonamides can be attributed to several mechanisms of action. These compounds have demonstrated the ability to inhibit kinases, crucial enzymes in cell signaling and growth, thereby disrupting cancer cell proliferation and survival. Additionally, they can lead to the formation of DNA damage and induce apoptosis through the activation of various cellular pathways^12,19–21^.

In this paper, we have investigated the cytotoxic/cytostatic activity of pyrazolo[4,3-*e*][1,2,4] triazine and pyrazolo[4,3-*e*]tetrazolo[1,5-*b*][1,2,4] triazine sulfonamide derivatives in CRC cells (DLD-1, HCT-116, and HT-29 cell lines) with different mutational status and sensitivity to chemotherapeutic agents in vitro. Furthermore, the effect of the addition of tetrazole to pyrazolo[4,3-*e*][1,2,4] triazine core and different terminal substituents (R) to phenylsulfonyl linker was explored in the context of compounds’ cytotoxic activity and selectivity for cancer cells. For this purpose, we have used two different methods – 3-(4,5-dimethylthiazol-2-yl)−2,5-diphenyltetrazolium bromide (MTT) assay and crystal violet assay to fully validate our findings. For the first time, the insights into the gastrointestinal safety of the most promising and lesser studied compounds were provided utilizing large intestine normal cells (CCD-841CoN cell line).

To fill the gap in the existing knowledge we have investigated the activity of four understudied pyrazolo[4,3-*e*]tetrazolo[1,5-*b*][1,2,4] triazines sulfonamide compounds (**MM134**, **−136**, **−137** and **− 139**) against three CRC cell lines (DLD-1, HCT-116 and HT-29) including their anti-proliferative (bromodeoxyuridine (BrdU) assay) and genotoxic (alkaline and neutral comet assays) properties. In the later step the compounds’ pro-apoptotic properties (dual acridine orange/ethidium bromide (AO/EB), Hoechst 33342/propidium ioide (HE/PI), annexin V- fluorescein isothiocyanate (FITC) staining) as well as their influence on mitochondrial membrane potential (ΔѰm) and the cell cycle were investigated in the most sensitive HCT-116 and HT-29 cell lines. Furthermore, the mechanism of the genotoxic properties of the compounds was studied using voltammetric techniques, providing insight into the potential of these heterocycles as important anti-cancer drug scaffolds. Finally, the effect of compounds on CRC spheroid cell viability/cell death was explored to favour the subsequent in vivo studies of these potential drug-like molecules.

## Materials and methods

### Synthesis and characterization of novel 1,2,4-triazine derivatives

**Fig. 1:**
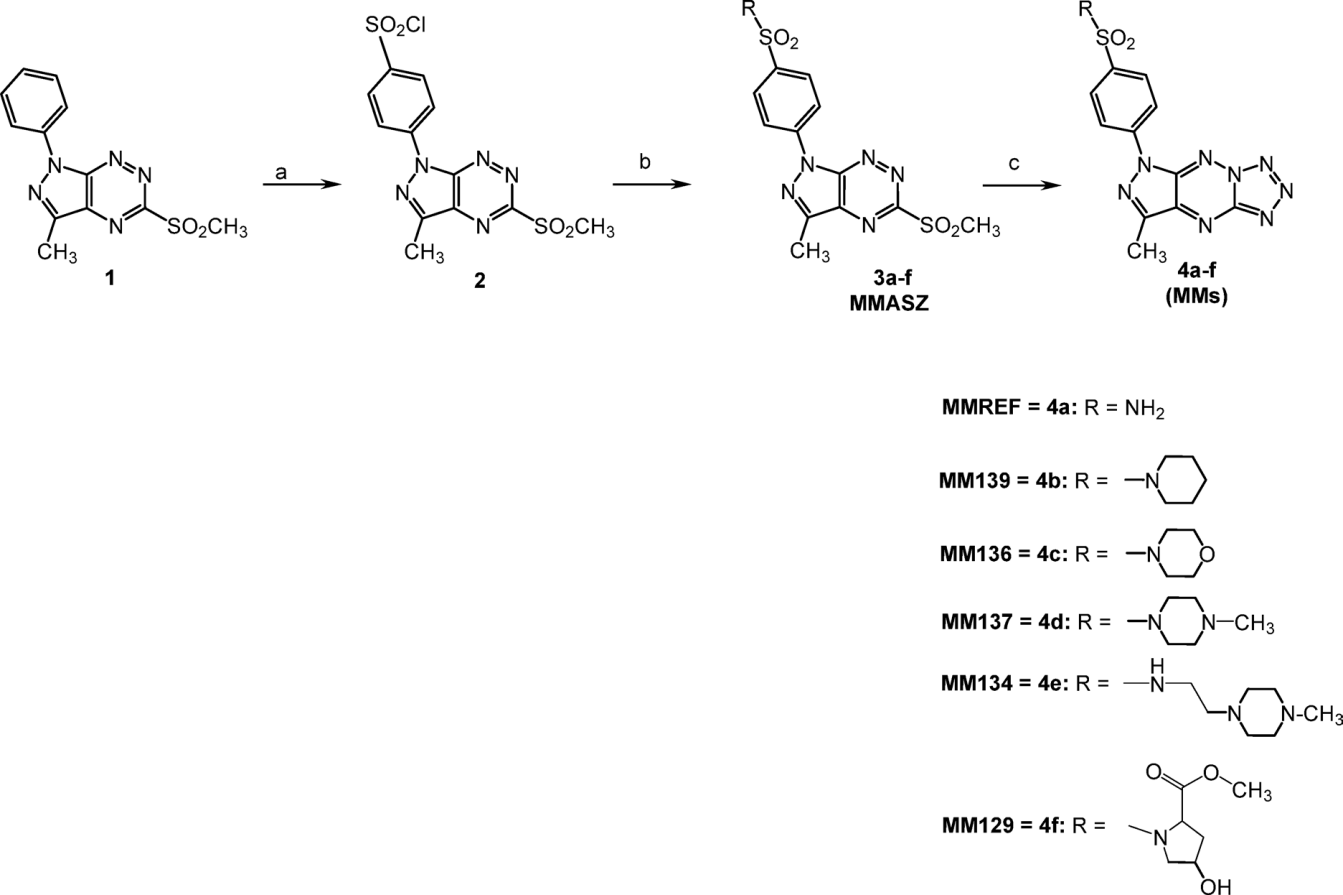
Synthesis of pyrazolo[4,3-*e*][1,2,4] triazine (**MMASZ**) and pyrazolo[4,3-*e*]tetrazolo[1,5-*b*][1,2,4] triazine sulfonamides (**MM**s). Reagents and conditions: **a** ClSO_3_H, 0 °C to rt, 2 h; **b** ammonia or appropriate amine derivatives, anhydrous MeCN, rt, stirred overnight; **c** NaN_3_, anhydrous EtOH, reflux, 18 h.

The target compounds **4a-f** (**MM** compounds) were achieved by a multi-step synthesis, starting from the known pyrazolotriazine **1** obtained using our previously published methods (Fig. 1)^22–24^. Briefly, the synthesis of the key derivative **2** was carried out based on the intramolecular nucleophilic substitution reaction of hydrogen at the 6-position of 1,2,4-triazine and oxidation of the SCH_3_ group under two-phase-transfer catalysis conditions^24^, followed by the use of the known chlorosulfonation reaction of the phenyl ring^25^. In the next step, compound **2** was reacted with ammonia or amine derivatives in anhydrous acetonitrile, stirring the reaction mixtures overnight at room temperature. The final tricyclic sulfonamides **4a-f** (**MM** compounds) were obtained by nucleophilic substitution of the methylsulfonyl group in compounds **3a-f** with sodium azide at the boiling point of anhydrous ethanol until the substrate disappeared from the reaction mixture (thin-layer chromatography (TLC) control of reaction progress)^23^. The compounds were isolated and purified using silica gel column chromatography and an appropriately selected eluent. The structure and purity of the newly synthesized compounds were characterized using ^1^H- and ^13^C-nuclear magnetic resonance (NMR) and high-resolution mass spectrometry (HRMS) methods, along with elemental analysis.

For detailed spectroscopic data, see the literature: (A) for compound **2**^23^, (B) for **3a** and **4a**^26^, (C) for **3b-e** and **4b-e**^27^, (D) for **MMASZ44** and **MM129**^28^.

### Biological studies

#### Chemicals

Cell culture media: RPMI-1640 and EMEM were purchased from Biowest (CytoGen, Poland) and American Type Culture Collection (ATCC, Rockville, USA), respectively. Fluorescent dyes: 4′,6-diamidino-2-phenylindole (DAPI), acridine orange (AO), ethidium bromide (EB), Hoechst 33,342 (HE), and propidium iodide (PI) were purchased from Sigma Aldrich Chemical Co. (USA). Reagents necessary to perform cell cultures: buffered saline (PBS), penicillin-streptomycin solution stabilized, fetal bovine serum (FBS), and trypsin-EDTA were purchased from Biowest (CytoGen, Poland). Reagents for cytotoxicity studies: (4,5-dimethylthiazol-2-yl)−2,3-diphenyltetrazolium bromide (MTT), crystal violet dye, dimethyl sulfoxide (DMSO), formaldehyde, and methanol were obtained from Sigma Aldrich Chemical Co. (USA). Chemotherapeutic agents: 5-fluorouracil (5-FU), bleomycin (B_10_), oxaliplatin (OXA), and 7-ethyl-10-hydroxycamptothecin (SN-38) were purchased from MedChemExpress LLC (TriMen Chemicals, Lodz, Poland). Comet assay reagents: normal melting point agarose (NMP), low melting point agarose (LMP), Triton X-100, sodium acetate (NaOAc), tris(hydroxymethyl)aminomethane (TRIS) were obtained from Sigma Aldrich Chemical Co. (USA). For cell proliferation assay: 5-bromo-2′-deoxyuridine (BrdU), bovine serum albumin (BSA), and Tween 20 were obtained from Sigma Aldrich Chemical Co (USA), fluoromount G was purchased from Invitrogen (Oxford, UK), normal goat serum was procured from Abcam (Cambridge, UK), and paraformaldehyde (PFA) was purchased from Polysciences, Inc. (Warrington, UK). Antibodies: Alexa Fluor 647 mouse anti-BrdU (BD Pharmingen, San Diego, CA, USA) primary antibody and the Alexa Fluor 594/488 goat anti-mouse (LifeTechnology, Warsaw, Poland) secondary antibody were employed in the BrdU assay. For cell cycle analysis: ethanol and RNAase A were purchased from Sigma Aldrich Chemical Co. (USA). For electrochemical studies: PBS and double-stranded salmon sperm DNA (dsDNA) were purchased from Sigma Aldrich Chemical Co. (USA). MitoTracker Red was purchased from Invitrogen (Waltham, MA, USA). Fluorescein isothiocyanate (FITC) – Annexin V Apoptosis Detection Kit I was purchased from B.D. Biosciences (Franklin Lakes, NJ, USA). For spheroid cell culture: Millicel ultra-low attachment plates, U-bottom, clear bottom were purchased from (Sigma Aldrich Chemical Co (USA)), and CellTiter-Glo Luminescent Cell Viability Assay was obtained from Promega (Promega Corporation, Madison, WI, USA).

#### Cell culture

HCT-116 (colorectal carcinoma, ATCC CCL-247), and HT-29 (colorectal adenocarcinoma, ATCC HTB-38) cancer cell lines and normal cell line CCD-841 CoN (large intestine normal cells, CRL-1790) were obtained from American Type Culture Collection (ATCC). DLD-1 (RRID: CVCL_0248) cell line was obtained from Horizon Discovery^29^. The MycoBlue Mycoplasma Detector kit, manufactured by Vazyme Biotech Co., Ltd. (Nanjing, China), was employed monthly to monitor and prevent mycoplasma contamination in the cell cultures.

DLD-1, HCT-116, and HT-29 cells were cultured in RPMI-1640 medium, and CCD-841 CoN cells were grown in EMEM medium, both supplemented 10% (v/v) FBS and 1% penicillin-streptomycin.

#### Cytotoxicity assays

##### Crystal violet assay

**Fig. 2:**
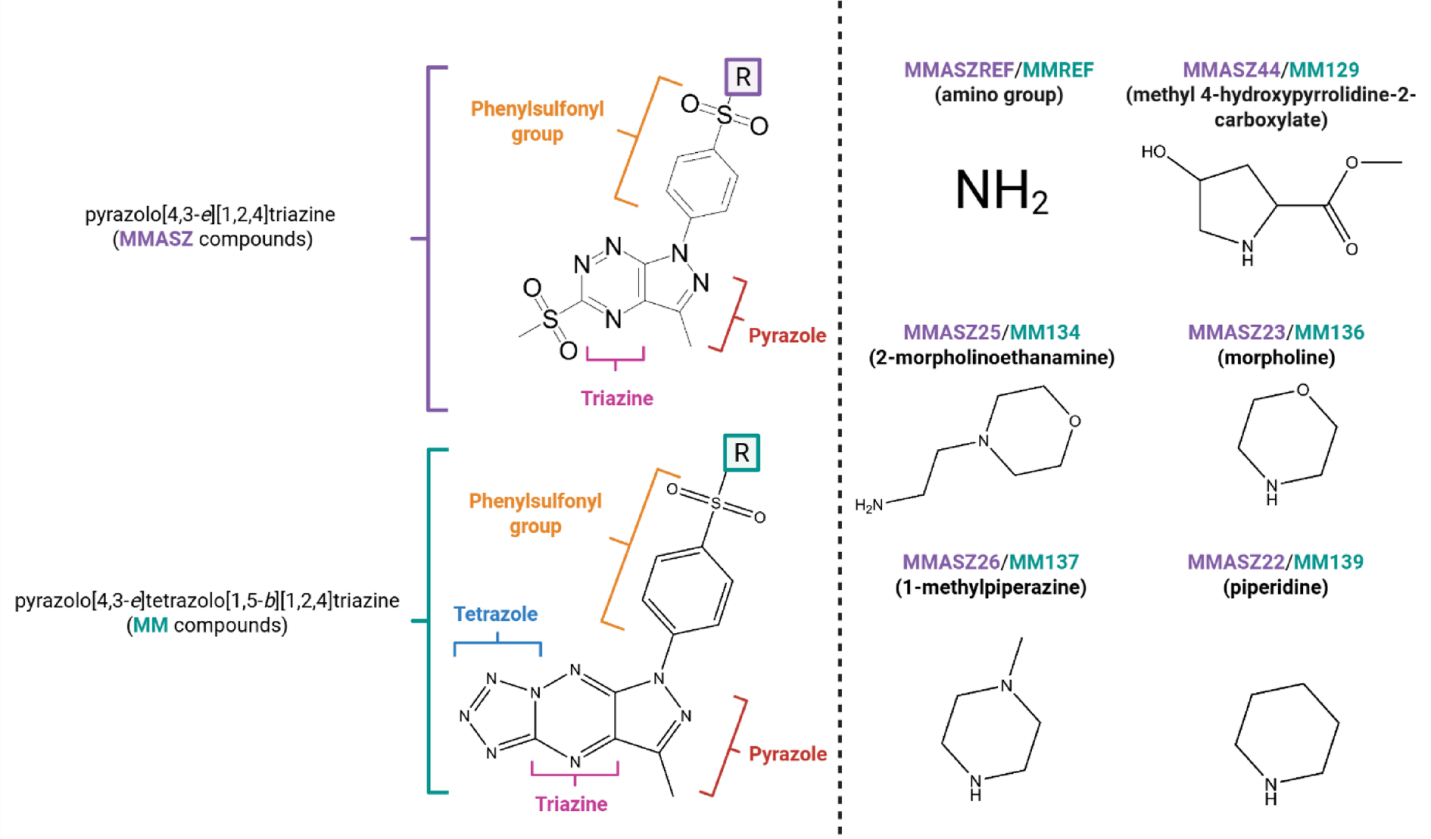
Chemical structures of investigated pyrazolo[4,3-*e*][1,2,4] triazine sulfonamides (**MMASZ** compounds) and pyrazolo[4,3-*e*]tetrazolo[1,5-*b*][1,2,4] triazine sulfonamides (**MM** compounds). Created in BioRender. Kciuk, M. (2026) https://BioRender.com/1hzmarw.

96-well cell culture plates were seeded at a density of 12 × 10^3^ (DLD-1, HCT-116, HT-29 cells) or 14 × 10^3^ (CCD-841 CoN) in 100 µL/well of appropriate cell medium. Following the placement of cell cultures in controlled conditions (37 °C; 5% CO_2;_ 24 h), cells were exposed to the tested compounds in the range of 0.05–50 µM. The structures of the compounds were provided in Fig. 2.

The concentrations were achieved through the process of diluting the compounds in DMSO and culture media, resulting in a final concentration of DMSO less than 0.5% v/v^30^. The experiment included non-treated controls and blanks (wells without cells). Following 24 h of incubation, the culture medium was removed, and the cells were washed with PBS (100 µL per well). After removing PBS, cells were fixed in 75% and then 100% methanol (50 µL per well) for 20 min each at 4 °C. The cells were then stained with 0.2% aqueous crystal violet solution (50 µL per well) for 20 min at room temperature. The crystal violet solution unbound to DNA was eluted by immersing the plates in water at room temperature. The plate was placed in a heater (37 °C) and left to dry. Then, the resulting precipitate was dissolved in DMSO (100 µL per well), and a spectrophotometric measurement was performed at a wavelength of 590 nm using a microplate reader (Power Wave XS BioTek Instruments, Inc., USA).

The obtained data were analyzed using GraphPad Prism 7 utility. A dose-response study was conducted to determine the 50% inhibitory concentration (IC_50_) of the investigated compounds. The IC_50_ value is the concentration of a tested substance that causes a 50% decrease in cell viability relative to the negative control, which is considered 100%. The cytotoxicity experiments were performed in duplicates.

$${\%}\ cell\ viability=\:\frac{\left(Absorbance\ value\ of\ treated\ cells\:-Absorbance\ value\ of\ blank\right)}{(Absorbance\:\ value\ of\ untreated\ cells-Absorbance\ value\ of\ blank)}\times\:100{\%}$$

##### MTT assay

MTT tetrazolium assay was used to re-evaluate the IC_50_ values obtained in the crystal violet assay. The cytotoxicity experiments were performed in duplicates. Cells were cultured and treated analogously to the above-described cytotoxicity assay used in our study. Additionally, 5-FU (concentration range 5–400) was used as a reference chemotherapeutic drug. Following 24-h incubation of cells with compounds, 20 µL of MTT tetrazolium salt (5 mg/mL in PBS) was added to each well, and plates were incubated for 3 h (37 °C; 5% CO2). The solutions were removed, and 100 µL of DMSO was added to dissolve formazan crystals. The absorbance reading was performed using a microplate reader, Power Wave XS, BioTek Instruments, Inc., USA at 570 nm.

Using data obtained from both the crystal violet assay and MTT assay, we have determined the consensus IC_50_ values as the mean cytotoxicity value obtained from the two assays. The selectivity index (SI) was calculated as the ratio of median consensus IC_50_ values obtained for the investigated **MMASZ**/**MM**-compounds in normal and cancer cell lines.

$$\:\mathrm{S}\mathrm{I}=\:\frac{\left(Mean\ IC50\ value\ calculated\ for\ normal\ cell\ lines\:\right)}{\left(Mean\ IC50\ value\ calculated\ for\ cancer\ cell\ lines\:\right)}$$

#### Bromodeoxyuridine (BrdU) assay

DLD-1, HCT-116, and HT-29 cells were seeded on the coverslips at a density of 3 × 10^4^ cells per 1 mL/well in 12-well plates. After a 24-h, the old medium was removed and replaced with 1 mL of fresh medium. The fresh medium contained **MM134**, **−136**, **−137**, and **− 139** compounds in concentrations representing the consensus IC_50_ values. Alternatively, 10 µM of OXA was used as a positive control. After 24 h, the solution containing compounds was supplemented with BrdU (10 µM/mL). Following 24 h, the medium was removed, and the cells were rinsed with 1 mL of PBS. The cells were then fixed in 70% cold ethanol (1 mL) and left at room temperature for 20 min. Afterward, the cells were rinsed twice with PBS (5 min, room temperature) and once with PBS containing 0.5% Triton X-100 (1 mL). The cells were treated with a mixture of 0.5 mL of PBS and 0.5 mL of 4 N HCl for 30 min at room temperature. Thereafter, the cells were rinsed twice with PBS and then placed in 1 mL of a 0.1 M sodium borate solution for 1 min at room temperature. The cells were rinsed with PBS once more and then exposed to a primary anti-BrdU antibody in a solution of PBS with 1% BSA and 0.5% Tween 20. This incubation step lasted for 1 h at room temperature in the dark. The cells were rinsed twice with a solution of PBS and 0.5% Tween 20 for 5 min. Afterward, the cells were incubated with a secondary antibody in a solution containing PBS, 1% BSA, and 0.5% Tween 20 for 1 h. The cells were rinsed twice with a solution of PBS containing 0.5% Tween 20, each time for 5 min. Subsequently, the cells were treated with DAPI at a concentration of 1 µg/mL for 15 min. Afterward, the cells were washed once with PBS at room temperature for 5 min. The coverslips were fixed in the drop of fluoromount G (mounting medium).

The experiment was conducted in triplicate, and a minimum of 500 cells per experiment were counted using a fluorescent microscope at a wavelength of 360 nm with the assistance of CellSens V2.3 software (Olympus, Tokyo, Japan). The data were provided as a percentage [%] ± SD of proliferating cells. An analysis of variance (ANOVA) was conducted, followed by Dunnett’s test to further investigate the discrepancies. A *p*-value below 0.05 was considered statistically significant (*p* < 0.05).

#### Alkaline/neutral comet assay

The comet assay is a sensitive and versatile method for detecting DNA damage at the level of individual cells. There are two main variants of the comet assay: the alkaline comet assay and the neutral comet assay, each serving different purposes in DNA damage assessment. The alkaline comet assay, developed by Singh et al. in 1988^31^, is performed under highly alkaline conditions (pH > 13). This version is particularly sensitive to DNA single-strand breaks (SSBs), alkali-labile sites, and DNA cross-linking. The neutral comet assay, conducted under neutral pH conditions, is specifically designed to detect double-strand breaks (DSBs). Developed as a complementary method to the alkaline version, it follows a similar procedure but maintains a neutral pH during electrophoresis. This ensures that only DSBs are detected, as SSBs do not cause significant DNA migration under these conditions^32–35^.

DLD-1, HCT-116, and HT-29 cells were seeded at a density of 1.5 × 10^5^ cells/mL onto 12-well plates. After 24 h, cells were treated with tested compounds at a concentration corresponding to 0.5xIC_50_ and IC_50_ consensus values obtained in the cytotoxicity tests or with Bleo (10 µM) as a positive control for another 24 h. Cells treated with a corresponding amount of DMSO not exceeding < 0.5% v/v were used as negative controls.

Alkaline and neutral comet assays were conducted as previously described^36,37^. Slides were stained with 1 µg/mL DAPI and coverslipped. DNA damage was evaluated using a fluorescence microscope at 360 nm with the use of CellSens (Olympus) software. Approximately 50 (*N* = 50) cells per slide were analyzed. The experiment was performed in duplicate. Data were represented as median tail DNA% with interquartile range and minimal and maximal values. The Kruskal-Wallis test was used to show a statistically significant (*p* < 0.05) difference between groups. Multiple comparisons using Dunn’s test were used. In all groups *N* > 200.

#### Apotosis studies

##### Dual acridine orange/ethidium bromide (AO/EB) and Hoechst 33342/propidium iodide (HE/PI) fluorescent staining

HCT-116 and HT-29 cells were seeded at a density of 1–1.5 × 10^5^ on 12-well plates. Following 24 h, cells were exposed to **MM**-compounds in consensus IC_50_ and 0.5 µM concentrations for 24- or 48 h. Following incubation time, cells were incubated with fluorochromes (AO/EB: 100 µM; 1:1, v/v) or (PI/HE: 1 µg/mL; 1:1, v/v) for 5 min at 37 °C in the dark. Cells were observed and counted in a fluorescence microscope (Olympus BX60 F5 Olympus Optical Co.Ltd) at 360 nm.

The fluorescence staining experiments were performed in triplicate and presented as mean percentage of necrotic and apoptotic cells [%] ± SD values. The differences between the experimental samples and the control sample were estimated by the ANOVA test followed by post-hoc by Dunnett’s test (*p* < 0.05, *N* = 200).

##### Flow cytometry analysis with annexin V-FITC/PI staining

The commercially available Annexin V-FITC Apoptosis Detection Kit was used to further explore the proapoptotic activity of **MM134**, **−136**, **−137**, and **− 139**. For this purpose HCT-116, and HT-29 cells were seeded at a density of 6–7 × 10^5^ on a 6-well plates in a volume of 2 mL medium. After 24-h incubation in controlled conditions (37 °C; 5% CO2), the cells were subjected to **MM** compounds at a consensus IC_50_ or 0.5 µM concentration. The experimental design included vehicle control (final solvent concentration was < 0.5% v/v DMSO) and positive compensation control (cells treated with 2 µM SN-38 – active metabolite of irinotecan). Cells were left for incubation for another 24 h (37 °C; 5% CO2). Upon exposure, cells were trypsinized and transferred to cytometric tubes, left for 40 min, and centrifuged at 1400 rpm for 10 min at 4 °C. Afterward, the supernatant was removed, and the precipitate was diluted in 1 mL of PBS. The rest of the procedure was performed according to the manufacturer’s instructions^27^.

The flow cytometry experiments were performed at least in duplicates and presented as mean percentage of necrotic and apoptotic cells ± SD values. The differences between the experimental samples and the control sample were estimated by the ANOVA test, followed by post-hoc by Dunnett’s test (*p* < 0.05, *N* > 10,000).

##### MitoTracker Red: mitochondrial membrane potential changes (ΔѰm)

MitoTracker Red is a fluorescent sensor that is frequently used to chemically identify mitochondria in living cells^38,39^. The accumulation and intensity of fluorescence correlate with the changes in mitochondrial transmembrane potential (MMP)^40^. HCT-116 and HT-29 cells were seeded at a density of 2 × 10^4^ on 96-well black clear-bottom plates. Following 24-h incubation in controlled conditions (37 °C; 5% CO_2_), cells were incubated with 0.5xIC_50_, consensus IC_50,_ or 2x IC_50_ concentrations of **MM134**, **−136**, **−137**, and − **139** for another 24 h. After 24 h, cells were incubated with MitoTracker Red (0.1 µM/200 µL PBS/well) for 40 min. Afterwards, MitoTracker Red solution was removed, and PBS (200 µL/well) was added. Fluorescence was read at an absorbance/emission of 581/644 nm using the SpectraMax i3x Multi-Mode Detection Platform. The experiment was performed in two biological replicates (*N* = 2), including a total of twelve technical replicates. Data were presented as a percentage of control fluorescence intensity [%] ± SD value. Dunnett’s test was used to represent statistically significant changes (*p* < 0.05) in the mean fluorescence intensity in treated samples compared with a negative control group.

#### Cell cycle analysis

Flow cytometry allows the identification of DNA content in cells after its staining with a fluorescent dye. One of the most common dyes used in molecular and cell biology is PI, which, upon permeabilization of cells with ethanol, binds with DNA through its intercalation and allows the estimation of cellular DNA content in different phases of the cell cycle^41^.

HCT-116 and HT-29 cells were seeded at a density of 6 × 10^5^ on a 6-well plate in a volume of 2 mL medium. After 24-h incubation in controlled conditions (37 °C; 5% CO_2_), the cells were subjected to **MM134**, **−136**, **−137**, and **− 139** compounds at a consensus IC_50_ or 0.5 µM concentrations. The experimental design included vehicle control (final solvent concentration was < 0.5% v/v DMSO). Cells were left for incubation for another 24 h (37 °C; 5% CO_2_).

After being exposed to **MM**-compounds, the cells were trypsinized and placed in cytometric tubes. They were then allowed to rest for 40 min before being centrifuged at 1400 rpm for 10 min at a temperature of 4 °C. After the supernatant was removed, the cell pellets were washed with PBS (1 mL) to purify the cells. After performing one more round of centrifugation of the cells, the supernatant was discarded, and the cells were resuspended in the remaining 200 µL of ice-cold PBS. After the suspensions were obtained, they were mixed before being put into fresh cytometric tubes that each contained 1 mL of ice-cold 70% ethanol. After centrifuging the cells in ethanol at 4500 rpm for 10 min at a temperature of 4 °C, the supernatant was discarded. Following incubation with a staining solution (250 µL/tube) for one hour in the dark at 37 °C, the cells were then subjected to analysis. The staining solution contained 5 µL of RNAase A (500 U/mL), 10 µL PI (500 µg/mL), and 870 µL of PBS.

The experiments were performed in triplicate and presented as mean percentage of cells in different cell cycle phases ± SD values. The differences between the experimental samples and the control sample were estimated by the ANOVA test followed by post-hoc by Dunnett’s test (*p* < 0.05).

#### Spheroid cell viability

To recapitulate the three-dimensional (3D) architecture and microenvironmental complexity of solid tumors more accurately than conventional two-dimensional (2D) monolayer cultures, we have employed spheroid cell culture to evaluate **MM129**, **MM134**, **MM136**, **MM137**, and **MM139** in this setting.

Cells were seeded on 96-well ultra-low attachment plates (ULAP) at a density of 5 × 10^3^ cells/100 µL per well. The plates were centrifuged at 200 g to allow aggregation of cells into a tight cluster (spheroid). Following 24 h, cells were treated for 6 days with the tested **MM**-compounds in 100 µL of fresh cell culture medium to achieve a final concentration of the compounds of 5 µM in each well. Afterwards, 100 µL of culture medium was removed and 100 µL of CellTiter-Glo 3D reagent was added to the cell culture medium present in each well. Thereafter, the plate was placed on a shaker to facilitate the spheroid lysis. The completeness of cell lysis was investigated under an inverted microscope, and the plate was incubated at room temperature for an additional 25 min to stabilize the luminescent signal before transferring 100 µL of cell lysate to an opaque white 96-well plate. Luminescence was recorded using a SpectraMax i3x Multi-Mode detection platform. ATP content in spheroid lysates was estimated and provided as relative luminescence units [RLU] ± SD. The decrease of luminescence reflects the higher abundance of dead cells in samples. The differences between the experimental and control groups were estimated by the ANOVA test, followed by post-hoc Dunnett’s test (* p = 0.0001, *N* = 4).

### Voltammetric studies

#### Apparatus and instrumentation

Electrochemical studies were performed using a potentiostat EmStat3 (PalmSens, the Netherlands), operated by the PS Trace software (version no. 5.9). In all measurements standard three-electrode system was used, with a platinum wire (Mineral, Poland) as an auxiliary electrode, Ag/AgCl/3MKCl electrode (Mineral, Poland) as a reference electrode and a boron-doped diamond electrode (Redoxme AB, Sweden, diameter: 2 mm) as a working electrode. The working electrode was mechanically polished with alumina slurry before each voltammetric measurement.

#### Electrochemical measurements

In the preliminary studies, both cyclic voltammetry (CV) and square-wave voltammetry (SWV) were applied to characterize the electrochemical behavior of the chosen sulfonamide derivatives (**MM134**, **−136**, **−137**, and **− 139**). The interaction between DNA and **MM**-compound was studied using SWV, as it is one of the most sensitive voltammetric techniques. In these studies, concentrations of **MM**-compounds were kept constant and analyzed in the presence and absence of dsDNA in PBS at pH 7.4. Before measurements, solutions were purged with argon for 5 min.

## Results

### Biological studies

#### Cytotoxicity assays

The cytotoxic activity and selectivity of pyrazolo[4,3-*e*][1,2,4]triazine compounds were evaluated against CRC cell lines (DLD-1, HCT-116, HT-29) and a normal colon epithelial cell line (CCD-841 CoN) using 24-h crystal violet and MTT assays (Tables S1 and S2). We have established a consensus IC_50_ value based on the results from both assays. Establishing a consensus helps minimize experimental errors and variability, leading to more reliable and credible data generated in further assays. Furthermore, we have calculated the SI (the ratio of the median consensus IC_50_ in normal cells to that in cancer cells) for each of the agents, including **MMASZ** (Table 1) and **MM** compounds (Table 2).

**Table 1:** Consensus IC_50_ ± SD [µM] and selectivity values derived from 24-h crystal violet and MTT assays for pyrazolo[4,3-*e*][1,2,4] triazine sulfonamides (**MMASZ** compounds). The selectivity index (SI) was calculated as the ratio of median consensus IC_50_ values obtained for the investigated **MMASZ** compounds in CDD-841 CoN normal cell line and DLD-1, HCT-116, and HT-29 cancer cell lines. N/D – indicates that the IC_50_ value could not be obtained in the 0.05–50 µM concentration range, N/S – indicates that the IC_50_ value established for one of the cancer cell lines was equal/higher than that obtained for a normal cell line, or both IC_50_ values obtained for cancer and normal cell lines were > 50 µM.

Cell line/		DLD-1			HCT-116			HT-29			CCD-841 CoN			
MMASZ compound	Moiety (*R*)	IC_50_ _(crystal violet)_	IC_50_ _(MTT)_	Consensus IC_50_	IC_50_ _(crystal violet)_	IC_50_ _(MTT)_	Consensus IC_50_	IC_50_ _(crystal violet)_	IC_50_ _(MTT)_	Consensus IC_50_	IC_50_ _(crystal violet)_	IC_50_ _(MTT)_	Consensus IC_50_	SI
**MMASZREF**	Amino group	13.03	6.3	**9.7 ± 4.8**	14.6	3.29	**8.9 ± 7.9**	> 50	> 50	**N/D**	> 50	> 50	> 50	**N/S**
**MMASZ44**	Methyl 4-hydroxypyrrolidine-2-carboxylate	18	> 30	**N/D**	15.18	10.54	**12.9 ± 3.28**	> 50	> 50	**N/D**	19.3	> 50	> 50	**N/S**
**MMASZ25**	2-morpholinoethanamine	2.62	2.52	**2.57 ± 0.07**	5.54	3.29	**4.4 ± 1.6**	7.31	3.55	**5.4 ± 2.7**	12.7	14.66	**13.7 ± 1.4**	**3.3**
**MMASZ23**	Morpholine	10.96	14.15	**12.56 ± 2.3**	3.73	10.03	**6.9 ± 4.5**	25.3	> 30	**N/D**	34.4	> 50	**N/D**	**N/D**
**MMASZ26**	1-methylpiperazine	6.1	11.7	**8.9 ± 3.9**	3.51	3.36	**3.4 ± 0.1**	8.45	3.28	**5.9 ± 3.7**	11.45	16.8	**14.1 ± 3.8**	**2.3**
**MMASZ22**	Piperidine	2.8	1.96	**2.38 ± 0.6**	1.82	3.48	**2.7 ± 1.2**	4.7	2.08	**3.4 ± 1.8**	9.24	3.25	**6.3 ± 4.2**	**2.2**

**Table 2:** Consensus IC_50_ ± SD [µM] and selectivity values derived from 24-h crystal violet and MTT assays for pyrazolo[4,3-*e*]tetrazolo[1,5-*b*][1,2,4] triazine (**MM** compounds). The selectivity index (SI) was calculated as the ratio of median consensus IC_50_ values obtained for the investigated **MM** compounds in CDD-841 CoN normal cell line and DLD-1, HCT-116, and HT-29 cancer cell lines. N/D – indicates that the IC_50_ value could not be obtained in the 0.05–50 µM concentration range, N/S – indicates that the IC_50_ value established for one of the cancer cell lines was equal/higher than that obtained for a normal cell line, or both IC_50_ values obtained for cancer and normal cell lines were > 50 µM.

Cell line/		DLD-1			HCT-116			HT-29			CCD-841 CoN			
MM compound	Moiety (*R*)	IC_50_ _(crystal violet)_	IC_50_ _(MTT)_	Consensus IC_50_	IC_50_ _(crystal violet)_	IC_50_ _(MTT)_	Consensus IC_50_	IC_50_ _(crystal violet)_	IC_50_ _(MTT)_	Consensus IC_50_	IC_50_ _(crystal violet)_	IC_50_ _(MTT)_	Consensus IC_50_	SI
**MMREF**	Amino group	0.85	0.58	**0.72** ± 0.19	0.38	0.33	**0.36** ± 0.04	0.8	0.74	**0.77** ± 0.42	2.78	1.69	**2.24** ± 0.8	**3.6**
**MM129**	Methyl 4-hydroxypyrrolidine-2-carboxylate	1.04	1.13	**1.1** ± 0.06	0.46	0.11	**0.29** ± 0.25	0.85	0.15	**0.5** ± 0.5	2.79	2.18	**2.5** ± 0.43	**3.9**
**MM134**	2-morpholinoethanamine	0.65 ± 0.03	0.89 ± 0.14	**0.77** ± 0.17	0.39 ± 0.06	0.38 ± 0.08	**0.385** ± 0.007	0.52 ± 0.07	0.22 ± 0.06	**0.37** ± 0.21	5.24 ± 0.5	2.48 ± 0.27	**3.86** ± 1.95	**7.7**
**MM136**	Morpholine	0.9 ± 0.06	0.42 ± 0.01	**0.66** ± 0.34	0.69 ± 0.06	0.29 ± 0.06	**0.49** ± 0.3	0.11 ± 0.01	0.13 ± 0.007	**0.12** ± 0.01	5.07 ± 0.42	2.65 ± 0.21	**3.86** ± 1.7	**9.2**
**MM137**	1-methylpiperazine	0.31	0.28 ± 0.007	**0.3** ± 0.02	0.25 ± 0.02	0.14 ± 0.007	**0.195** ± 0.08	0.21 ± 0.01	0.12	**0.165** ± 0.06	1.52 ± 0.08	1.16 ± 0.02	**1.34** ± 0.25	**6.1**
**MM139**	Piperidine	0.27 ± 0.07	0.87 ± 0.03	**0.57** ± 0.42	0.2 ± 0.02	0.28 ± 0.1	**0.24** ± 0.06	0.33 ± 0.19	0.17 ± 0.04	**0.25** ± 0.11	2.62 ± 0.6	1.59 ± 0.44	**2.1** ± 0.73	**6**

**MMASZ25** (bearing 2-morpholinoethanamine) showed the highest selectivity among pyrazolo[4,3-*e*][1,2,4]triazine sulfonamides (SI = 3.3), with low IC_50_ values in cancer cells (2.57 ± 0.07, 4.4 ± 1.6, and 5.4 ± 2.7 µM for DLD-1, HCT-116, and HT-29 cells, respectively) and relatively higher IC_50_ in normal cells (13.7 ± 1.4 µM). **MMASZ26** with 1-methylpiperazine moiety (SI = 2.3) and **MMASZ22** with piperidine moiety (SI = 2.2) exhibited some cancer selectivity but were less selective than **MMASZ25** and exhibited similar cytotoxicity towards cancer cells (Table 1).

In contrast, among the pyrazolo[4,3-*e*]tetrazolo[1,5-*b*][1,2,4]triazine sulfonamides, **MM136** (bearing morpholine) and **MM134** (bearing 2-morpholinoethanamine) demonstrated the highest selectivity, with SI values of 9.2 and 7.7, respectively, indicating preferential cytotoxicity towards cancer cells. **MM137** (1-methylpiperazine) and **MM139** (piperidine) also exhibited favorable selectivity (SI = 6.1 and 6, respectively). In comparison, the reference compounds **MMREF** and **MM129** displayed a lower SI of 3.6 and 3.9, suggesting that specific structural modifications, such as the introduction of a morpholine and N-aminoethylolmorpholine moiety, enhance anticancer selectivity (Table 2). Activity of **MM**-compounds exceeded the cytotoxic activity of 5-FU (50% viability reduction was observed in concentrations higher than 50 µM) (**Figure S1**). Nevertheless, the selectivity of compounds could not be compared. Examples of dose-response curves obtained in the MTT assay for **MM134**, **−136**, **−137**, and **− 139** compounds in investigated cell lines were provided in supplementary material: Figures S2-S5. Other dose-response data are available on request from the authors of this work.

####  Bromodeoxyuridine (BrdU) assay

**Fig. 3:**
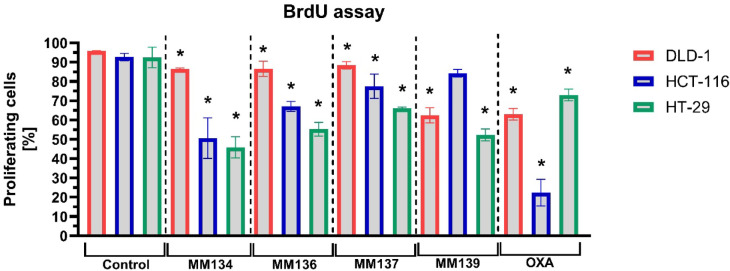
DLD-1, HCT-116, and HT-29 cell proliferation following treatment of cells with **MM**-compounds (**MM134**, **−136**, **−137**, and **− 139**) used in consensus IC_50_ concentrations derived from 24-h crystal violet and MTT assay. 10 µM oxaliplatin (OXA) was used as a positive control. Data were presented as a mean percentage [%] of proliferating cells ± SD. Dunnett’s test was used to represent statistically significant changes (*p* < 0.05) in the mean % of proliferating cells in samples compared with a negative control (indicated with a * mark).

To investigate whether the observed anticancer effect is attributed to the inhibition of cell proliferation or the direct cytotoxic impact we have performed a BrdU incorporation assay following incubation of DLD-1, HCT-116, and HT-29 cells with **MM134**, **−136**, **−137** and **− 139** compounds compounds used in consensus IC_50_ concentrations and OXA (10 µM) used in the experiment as a positive control (Fig. 3).

In the DLD-1 cell line, each of the **MM**-compounds exhibited antiproliferative activity: **MM134** (proliferating cells = 86.5 ± 0.6%), **MM136** (proliferating cells = 86.6 ± 3.9%), **MM137** (proliferating cells = 88.5 ± 1.9%) and **MM139** (proliferating cells = 63 ± 3%) compared to negative control (proliferating cells = 95.8 ± 0.3%) (*p* < 0.05). At the same time, **MM139** exhibited higher cytostatic properties than 10 µM OXA (proliferating cells = 63 ± 3%).

In contrast, in HCT-116 cells, only the **MM139** compound has not induced a statistically significant decrease in the fraction of proliferating cells compared to the negative control (proliferating cells = 92.8 ± 1.9%). The antiproliferative activity increased in order of **MM137** (proliferating cells = 77.6 ± 6.4%; *p* < 0.0001), **MM136** (proliferating cells = 67.1 ± 2.6; *p* = 0.0008) and **MM134** (proliferating cells = 50.7 ± 10.6; *p* = 0.0339). Nevertheless, the activity have surpassed the effects of OXA (proliferating cells = 22.4 ± 6.9%).

The strongest inhibitory effects (*p* < 0.0001) were observed in HT-29 cells where **MM134** (% of (proliferating cells = 45.9 ± 5.5%), **MM136** (proliferating cells = 55.3 ± 3.5%), **MM137** (proliferating cells = 66.06 ± 0.6%), **MM139** (proliferating cells = 52.4 ± 3%) exceeded the anti-proliferative activity of OXA (proliferating cells = 73 ± 3%; *p* = 0.0002) compared to control group (proliferating cells = 92.5 ± 5.3%).

#### Alkaline comet assay

**Fig. 4:**
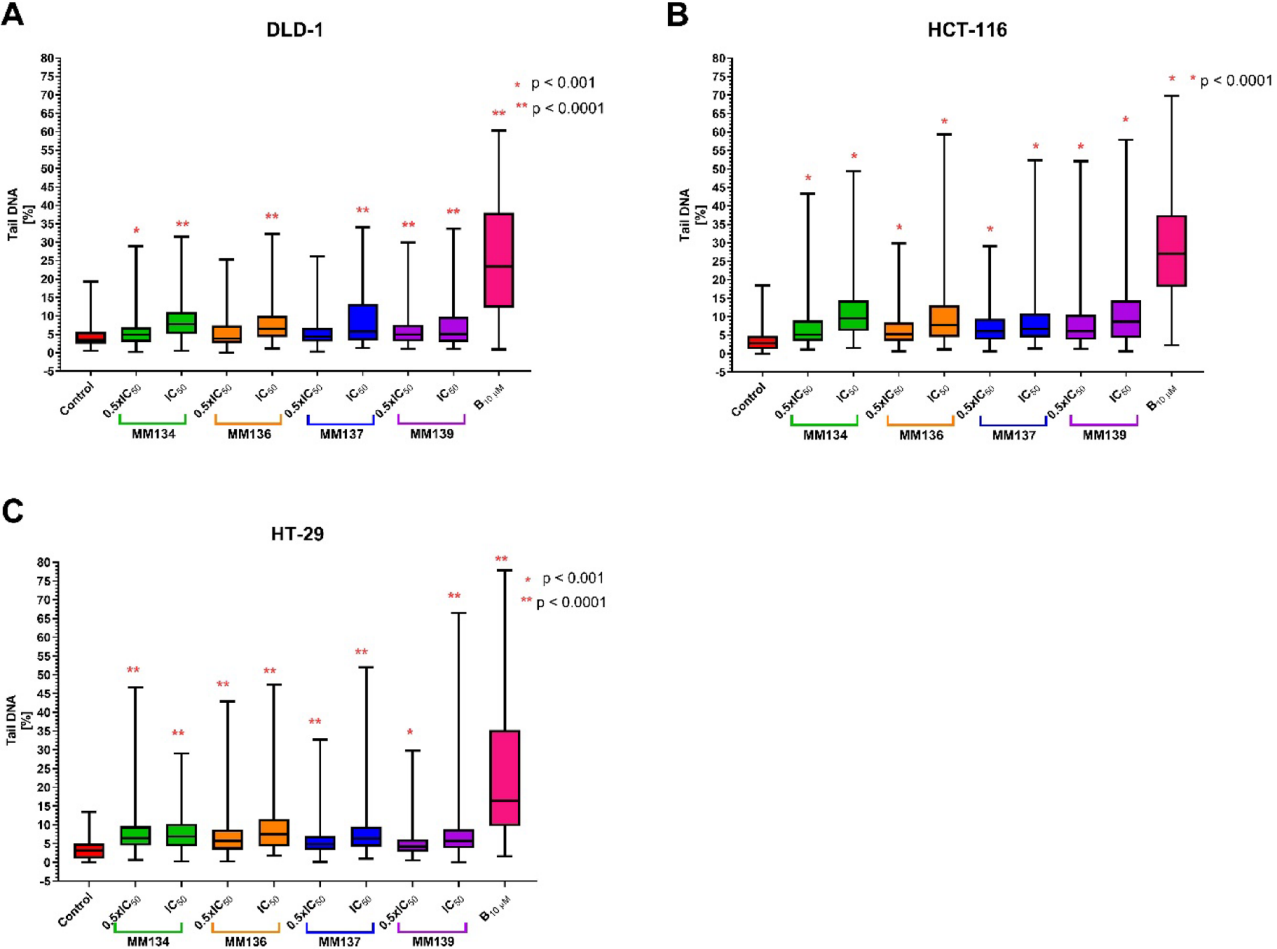
DNA damage in **A** DLD-1, **B** HCT-116, and **C** HT-29 cells following 24-h incubation with 0.5xIC_50_ and IC_50_ concentrations of **MM134**, **−136**, **−137**, and **− 139**, and 10 µM bleomycin (B_10µM_; positive control) assessed using alkaline comet assay. Data were presented as median tail DNA% with interquartile range and minimal and maximal values. The Kruskal-Wallis test was used to show a statistically significant (*) difference between groups. Multiple comparisons using Dunn’s test were used (*p* < 0.05, *N* = 200).

Next, we analyzed DNA damage using alkaline and neutral comet assays by utilizing CASP: Comet Assay Software Project Lab (http://casplab.com) software following incubation of DLD-1, HCT-116, and HT-29 cells with the tested **MM** compounds for 24-h^42^ (Fig. 4A-C). The data were displayed as the median percentage of comet tail DNA, along with the interquartile range and the minimum and maximum values.

In DLD-1 cells **(A)**, all of the **MM**-compounds used in respective IC_50_ concentrations induced statistically significant (*p* < 0.0001) increase in DNA damage expressed as median tail DNA % which equaled 7.8% (**MM134**), 6.5% (**MM136**), 5.8% (**MM137**) and 5.1% (**MM139**) and have not exceeded this of 10 µM bleomycin, which equaled 23.4% (**B**_**10 µM**_) as compared to negative control (median tail DNA% = 3.5).

In HCT-116 cells **(B)**, **MM**-compounds used in both 0.5xIC_50_ and IC_50_ concentrations induced statistically significant (*p* < 0.0001) increase in DNA damage compared to the negative control (median tail DNA% = 2.8%), which equaled 5.2% (0.5xIC_50_ **MM134**), 9.6% (IC_50_ **MM134**), 5.3% (0.5xIC_50_ **MM136**), 7.7% (IC_50_ **MM136**), 6.1% (0.5xIC_50_ **MM137**), 6.7% (IC_50_ **MM137**), 6.1% (0.5xIC_50_ **MM139**) and 8.6% (IC_50_ **MM139**). Furthermore, **B**_**10µM**_ also induced a statistically significant (*p* < 0.0001) increase in DNA damage (median tail DNA% = 27.05) compared to the negative control.

Similarly to HCT-116 cells, in HT-29 cells **(C) MM**-compounds used in both 0.5xIC_50_ and IC_50_ concentrations induced statistically significant (*p* < 0.0001) increase in DNA damage compared to the negative control (median tail DNA% = 3.2), which equaled 6.4% (0.5xIC_50_ **MM134**), 6.9% (IC_50_ **MM134**), 5.7% (0.5xIC_50_ **MM136**), 7.5% (IC_50_ **MM136**), 4.8% (0.5xIC_50_ **MM137**), 6.3% (IC_50_ **MM137**), 4.2% (0.5xIC_50_ **MM139**) and 5.7% (IC_50_ **MM139**). DNA damage has not exceeded that induced by positive control (**B**_**10 µM**_; median tail DNA% = 16.4). Examples of comet images obtained in an alkaline comet assay were included in the (Supplementary material: Figure S6-9).

#### Neutral comet assay

**Fig. 5:**
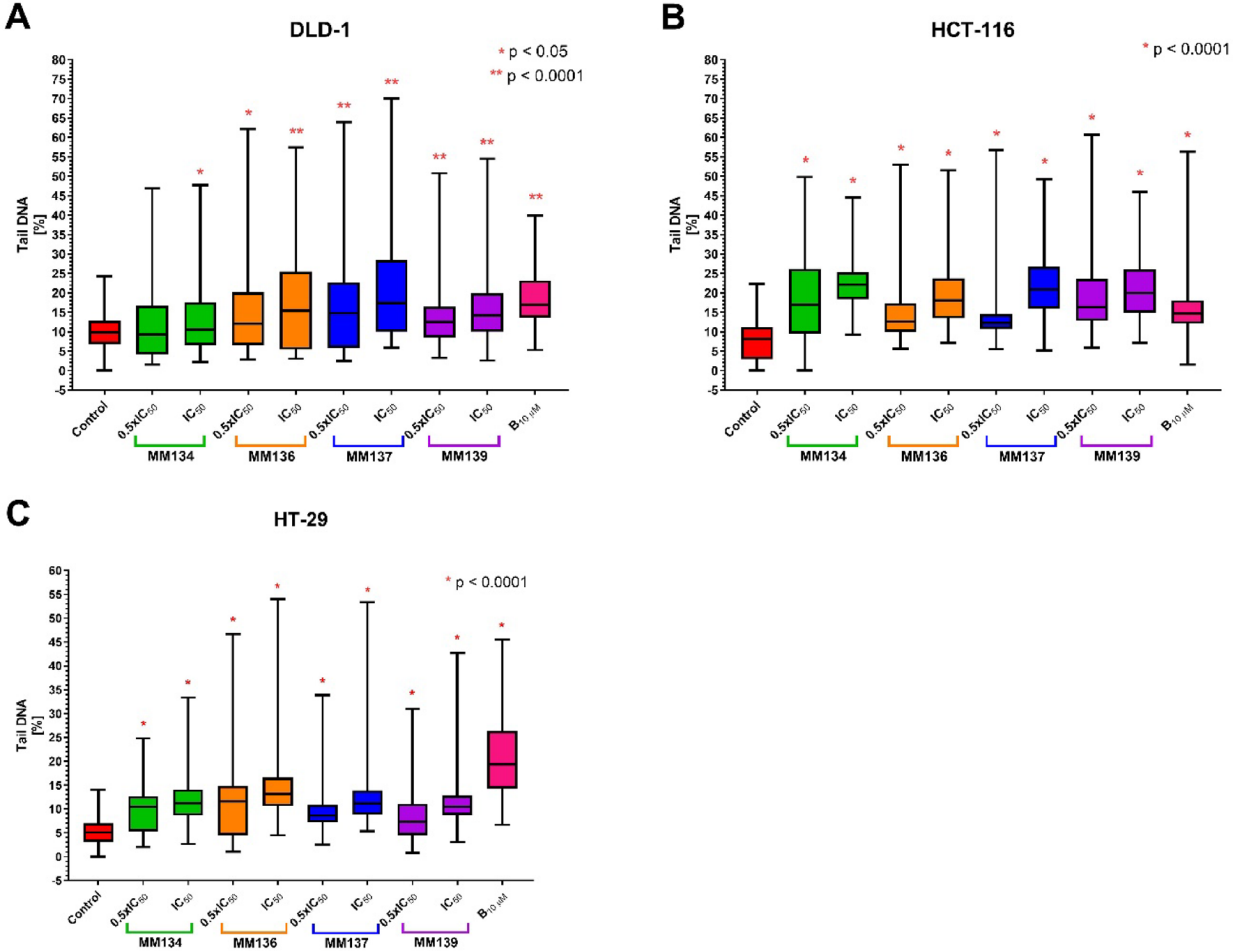
DNA damage (double-strand breaks; DSBs) in **A** DLD-1, **B** HCT-116, and **C** HT-29 cells following 24-h incubation with 0.5xIC_50_ and IC_50_ concentrations of **MM134**, **−136**, **−137**, **−139**, and 10 µM bleomycin (B_10µM_; positive control) evaluated using neutral comet assay. Data were represented as median tail DNA% with interquartile range and minimal and maximal values. The Kruskal-Wallis test was used to show a statistically significant (*) difference between groups. Multiple comparisons using Dunnett’s test were used. In all groups, *N* > 200.

To further explore the mechanism of genotoxic activity of the **MM**-compounds, a neutral comet assay was performed in DLD-1 **(A)**, HCT-116 **(B)**, and HT-29 **(C)** CRC cells (Fig. 5).

In DLD-1 cells **(A)**, **MM**-compounds used in IC_50_ concentrations led to a statistically significant (*p* < 0.05) increase in DSBs prevalence compared to the negative control (median tail DNA% = 9.8). DNA damage, as evidenced by neutral comet assay, represented as the median tail DNA%, equaled 10.5%, 15,4%, 17,4%, and 14.1% for **MM134**, **MM136**, **MM137**, and **MM139**, respectively. **MM137** used in IC_50_ concentration exceeded the DNA-damaging potential of the positive control (**B**_**10 µM**_; median tail DNA% = 16.9).

In HCT-116 cells **(B)**, all of the tested compounds in both 0.5xIC_50_ and IC_50_ concentrations induced statistically significant (*p* < 0.0001) increase in median tail DNA % parameter when compared to the negative control (median tail DNA% = 8.1) and equaled 16.9% (0.5xIC_50_ **MM134**), 22.1% (IC_50_ **MM134**), 12.6% (0.5xIC_50_ **MM136**), 18% (IC_50_ **MM136**), 12.2% (0.5xIC_50_ **MM137**), 20.9% (IC_50_ **MM137**), 16.3% (0.5xIC_50_ **MM139**) and 19.9% (IC_50_ **MM139**). **MM134** and **− 9** used in both investigated concentrations, and **MM136/137** used in IC_50_ concentration, surpassed the DSBs formation induced by the positive control (**B**_**10 µM**_; median tail DNA% = 14.7%).

Similarly in HT-29 cells **(C)**, all of the tested compounds in both 0.5xIC_50_ and IC_50_ concentrations induced statistically significant (*p* < 0.0001) increase in median tail DNA % compared to the negative control (median tail DNA% = 5.1) and were: 10.5% (0.5xIC_50_ **MM134**), 11.1% (IC_50_ **MM134**), 11.6% (0.5xIC_50_ **MM136**), 13.1% (IC_50_ **MM136**), 8.6% (0.5xIC_50_ **MM137**), 11.1% (IC_50_ **MM137**), 7.3% (0.5xIC_50_ **MM139**) and 10.5% (IC_50_ **MM139**). However, tested compounds have not exceeded the number of DSBs induced by the positive control (**B**_**10 µM**_; median tail DNA% = 19.3). In all cancer cell lines, DNA damage increased with the rise in the concentration of the tested **MM**-compound, which is a characteristic feature of genotoxic compounds. Examples of comet images obtained in a neutral comet assay for HCT-116 cells were included in the (Supplementary material: Figure S8).

#### Apoptosis studies

The utilization of apoptotic staining methods (AO/EB, HE/PI)^43–45^, detection of phosphatidylserine (PS) exposition during apoptosis using annexin V-FITC^46^, and evaluation of mitochondrial membrane potential (ΔѰm)^47,48^ changes is essential for a comprehensive evaluation of compounds’ mechanism of action. All these assays were used to determine cell death in HCT-116 and HT-29 cells following treatment with **MM134**, **−136**, **−137**, and **− 139** compounds at IC_50_ concentrations (Table 3).

**Table 3:** Pro-apoptotic properties of pyrazolo[4,3-*e*]tetrazolo[1,5-*b*][1,2,4] triazine sulfonamides (**MM** compounds) in HCT-116 and HT-29 cells using dual acridine orange/ethidium bromide (AO/EB), Hoechst 33,342/propidium iodide (HE/PI) fluorescent staining and flow cytometry analysis with annexin V-FITC/PI staining. Results were presented as a mean percentage [%] ± SD of apoptotic cells in cells treated with IC_50_ concentrations of **MM134**, **−136**, **−137**, and **− 139** compounds. Dunnett’s test was used to represent statistically significant changes (*p* < 0.05) in the mean % of apoptosis in treated samples compared with a negative control. Statistically significant changes in apoptotic cell fraction were indicated in bold.

Apoptosis detection method	Cell line	Incubation time	Control	MM134	MM136	MM137	MM139
Acridine orange/ethidium bromide (AO/EB) staining	HCT-116	24-h	0.66 ± 0.3	**23.17 ± 0.8**	**28.7 ± 1.5**	**26.7 ± 1.5**	**17 ± 2**
		48-h	6.33 ± 1.53	**29 ± 3**	**62.7 ± 3**	**33.7 ± 4**	**28.7 ± 1.5**
	HT-29	24-h	1.2 ± 0.8	**12.3 ± 2.5**	**3.16 ± 0.8**	**10 ± 1**	5 ± 2.6
		48-h	9.13 ± 2.2	**34.7 ± 5.7**	**31.8 ± 5.4**	**41.3 ± 5.5**	23.7 ± 4.16
Hoechst 33342/propidium ioide (HE/PI) staining	HCT-116	24-h	1.6 ± 0.4	**30.3 ± 2.5**	**30.7 ± 2**	**30 ± 2**	**11.7 ± 1.5**
		48-h	10.8	**36.7 ± 3.5**	**50.3 ± 1.5**	**48 ± 2.7**	**23.3 ± 3**
	HT-29	24-h	3.5 ± 1.3	**10.3 ± 1.5**	4.2 ± 0.8	**10.3 ± 0.6**	**10.7 ± 1.2**
		48-h	13.7 ± 1.5	**30.7 ± 3**	**31.8 ± 2.2**	**32.7 ± 3**	**26 ± 2**
Flow cytometry analysis with annexin V-FITC/PI staining	HCT-116	24-h	5.2 ± 0.3	**13.9 ± 2.8**	**24.3 ± 1.9**	**13.7 ± 2**	**13.7 ± 1.7**
		48-h	5.5 ± 0.7	**27.5 ± 2.12**	**64 ± 2.8**	**31.5 ± 2.12**	**28 ± 1.4**
	HT-29	24-h	15.8 ± 0.1	**8.8 ± 1.2**	11.2 ± 2.4	**10.2 ± 2.2**	**9.8 ± 0.4**
		48-h	9.2 ± 2.5	**37 ± 4.6**	**33.2 ± 5.5**	**39.5 ± 5.2**	23 ± 4.6

Results demonstrated a time-dependent increase in apoptosis, with higher apoptotic cell percentages observed after 48 h of treatment compared to 24 h. Among the tested compounds, **MM136** consistently induced the highest levels of apoptosis in HCT-116 cells. Using AO/EB staining, the apoptotic fraction percentage for **MM136** reached 62.7 ± 3% after 48 h, significantly higher than the control (6.33 ± 1.53%, *p* = 0.0047). Similarly, HE/PI staining and flow cytometry confirmed robust apoptotic induction by **MM136**, with apoptotic percentages of 50.3 ± 1.5% and 64 ± 2.8%, respectively, after 48 h (*p* < 0.05). **MM137** demonstrated comparable activity, with apoptotic fraction ranging from 31.5 ± 2.12% (flow cytometry) to 48 ± 2.7% (HE/PI staining) (*p* < 0.05) for 48 h incubation. In contrast, **MM134** and **MM139** showed moderate apoptotic effects, with **MM139** being the least effective.

In HT-29 cells, the pro-apoptotic effects of the compounds were less pronounced. **MM136** and **MM137** displayed the highest activity, particularly at 48-h, with apoptotic percentages of 31.8 ± 5.4% and 41.3 ± 5.5% (AO/EB staining), 31.8 ± 2.2% and 32.7 ± 3% (HE/PI staining), and 33.2 ± 5.5% and 39.5 ± 5.2% (flow cytometry), respectively (*p* < 0.05).

In addition to the pro-apoptotic properties, the above-mentioned methods also allow the estimation of necrotic properties of compounds. The statistically significant (*p* < 0.05) increase in necrotic cell fraction was observed only in HT-29 cells following 24-h incubation for **MM136** and **MM137** compounds in HE/PI staining (25.3 ± 1.5% and 19.3 ± 1.2% respectively; *p* < 0.05) and 48-h incubation in AO/EB staining (14.6 ± 2.4% and 20 ± 2% respectively; *p* < 0.05). In contrast, flow cytometry analysis revealed no increase in necrotic cell fraction following both 24- and 48-h incubation of HCT-116 and HT-29 cells with tested compounds, except for 24-h incubation of HCT-116 cells with the **MM136** compound (% of necrotic cells = 8.5 ± 1.7; *p* = 0.0152) (Table S3).

Furthermore, to allow easier comparison of the pro-apoptotic (Table S4) and pro-necrotic (Table S5) properties of the compounds, we have evaluated their activity in one selected concentration (0.5 µM). Among the tested compounds, **MM137** exhibited the highest apoptotic induction, with apoptosis levels reaching 85 ± 3% (AO/EB), 86 ± 1.7% (HE/PI), and 83.5 ± 2.12% (annexin V-FITC/PI) in HCT-116 cells at 48 h. **MM136** also demonstrated strong pro-apoptotic effects, with apoptosis rates between 63 ± 1.7% and 77.5 ± 2.8% in HCT-116 and 33.2 ± 5.5% to 69.3 ± 9.9% in HT-29 cells across different assays for 48-h incubation. **MM134** exhibited moderate activity, while **MM139** showed the weakest apoptotic response, with a maximum of 47.3 ± 2% apoptosis in HCT-116 and 36.7 ± 2% in HT-29 at 48 h. Notably, the apoptotic effects were more pronounced in HCT-116 cells than in HT-29, indicating potential cell-line-dependent sensitivity.

Furthermore, the results demonstrated that necrosis induction varied across compounds, incubation times, and cell lines. AO/EB staining revealed that **MM137** exhibited the highest necrotic response in HCT-116 cells at 24 h (35.7 ± 3.5%), while **MM136** and **MM134** also significantly increased necrosis (23 ± 3% and 26.3 ± 3.2%, respectively). However, at 48 h, necrosis levels declined, particularly for **MM136** (6.4 ± 0.6%). In HT-29 cells, no statistically significant increase in necrotic cell fraction was observed for 24-h incubation, while **MM134** (20.3 ± 2.4%), **MM137** (23.9 ± 1.7%), and **MM139** (19.3 ± 2%) induced significant necrosis at 48 h. Similar trends were observed with HE/PI staining, where **MM137** induced the highest necrosis in HCT-116 cells at 24 h (22.7 ± 2.5%), and **MM134** exhibited the strongest necrotic effect in HT-29 cells (31 ± 3.6%). No statistically significant changes in necrotic cell fraction were observed in flow cytometric analysis following 24-h incubation of cells with tested compounds, while the **MM134** compound showed the highest necrotic response in both HCT-116 (% of necrotic cells = 21.5 ± 2) and HT-29 cells (% of necrotic cells = 24.4 ± 2) following 48-h incubation of respective cells.

**Fig. 6:**
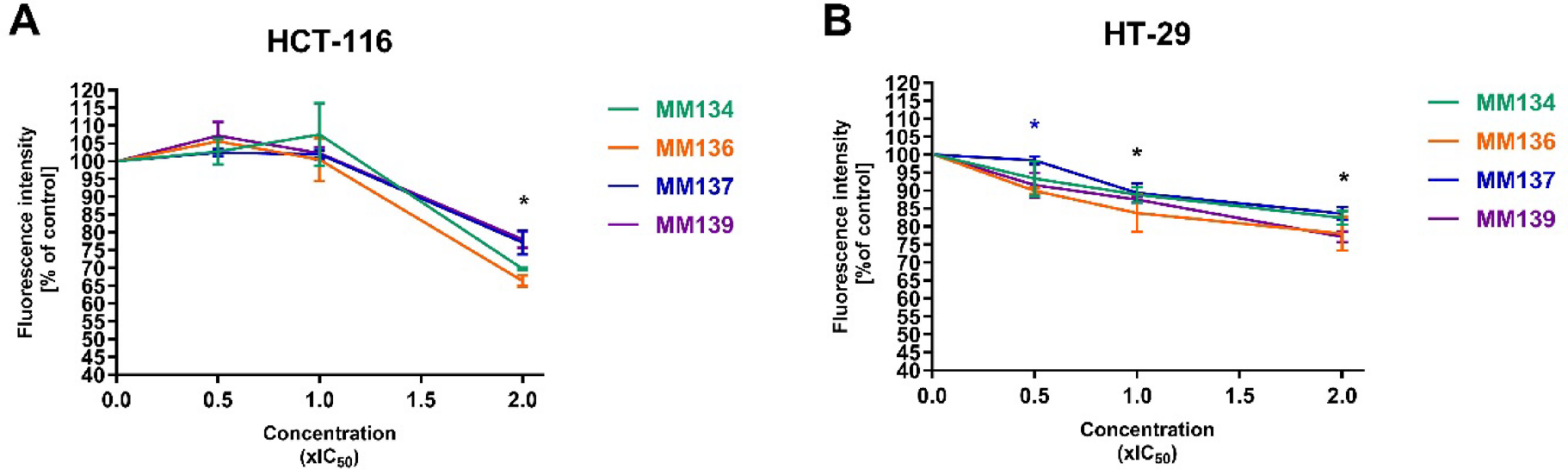
Changes in the mitochondria membrane potential (MMP) in response to treatment of **A** HCT-116, and **B** HT-29 cells with **MM134**, **−136**, **−137**, and **− 139**. Data were presented as a percentage of control fluorescence intensity [%] ± SD value. Dunnett’s test was used to represent statistically significant changes (*p* < 0.05) in the mean fluorescence intensity in treated samples compared with a negative control, indicated as an asterisk (*).

As a consequence of the change in the MMP, Mitotracker Red is deposited in the mitochondrial matrix, and its fluorescence intensity serves as a reflection of the state of the mitochondria. The effect of **MM134**, **−136**, **−137**, and **− 139** on MMP used in concentrations of 0.5xIC_50_, IC_50,_ and 2xIC_50_ was estimated following 24-h incubation of HCT-116 and HT-29 with **MM**-compounds. A decrease in the fluorescence intensity accompanied the increase in compounds’ concentration **(**Fig. 6**)**.

The strongest reduction of fluorescence intensity following 24-h incubation of cells with **MM**-compounds was observed for the highest (2xIC_50_) concentration of **MM136** compound in the HCT-116 cell line, where the mean % of control fluorescence equaled 66.63 ± 1.53% (*p* = 0.001).

In the HT-29 cell line, **MM136** and **MM137** exhibited similar potential for MMP reduction (the mean % of control fluorescence was 78 ± 4.69% (*p* = 0.008) and 77.19 ± 1.49% (*p* = 0.0015) for 2xIC_50_ concentrations, respectively).

#### Cell cycle analysis

During the cell cycle, the amount of DNA in the S-phase increases as a consequence of DNA replication. During the G_2_/M phase of the cell cycle, when there are two complete copies of the DNA, the cell divides into two new cells. Therefore, cells that are at various phases of the cell cycle have different amounts of DNA. In contrast, due to the partial degradation of DNA during the process of apoptosis, the amount of DNA in these cells is decreased. The DNA in cells can be stained with fluorescent dyes, including widely used PI, which intercalates the DNA molecule. The fluorescence signal is proportional to the PI bound to DNA in cells. Apoptotic cells stained with PI and examined with a flow cytometry exhibit a broad hypodiploid (sub-G_1_) peak, which can be easily distinguished from the slender peak of cells with normal (diploid) DNA content in the red fluorescence channels^41,49^.

**Fig. 7:**
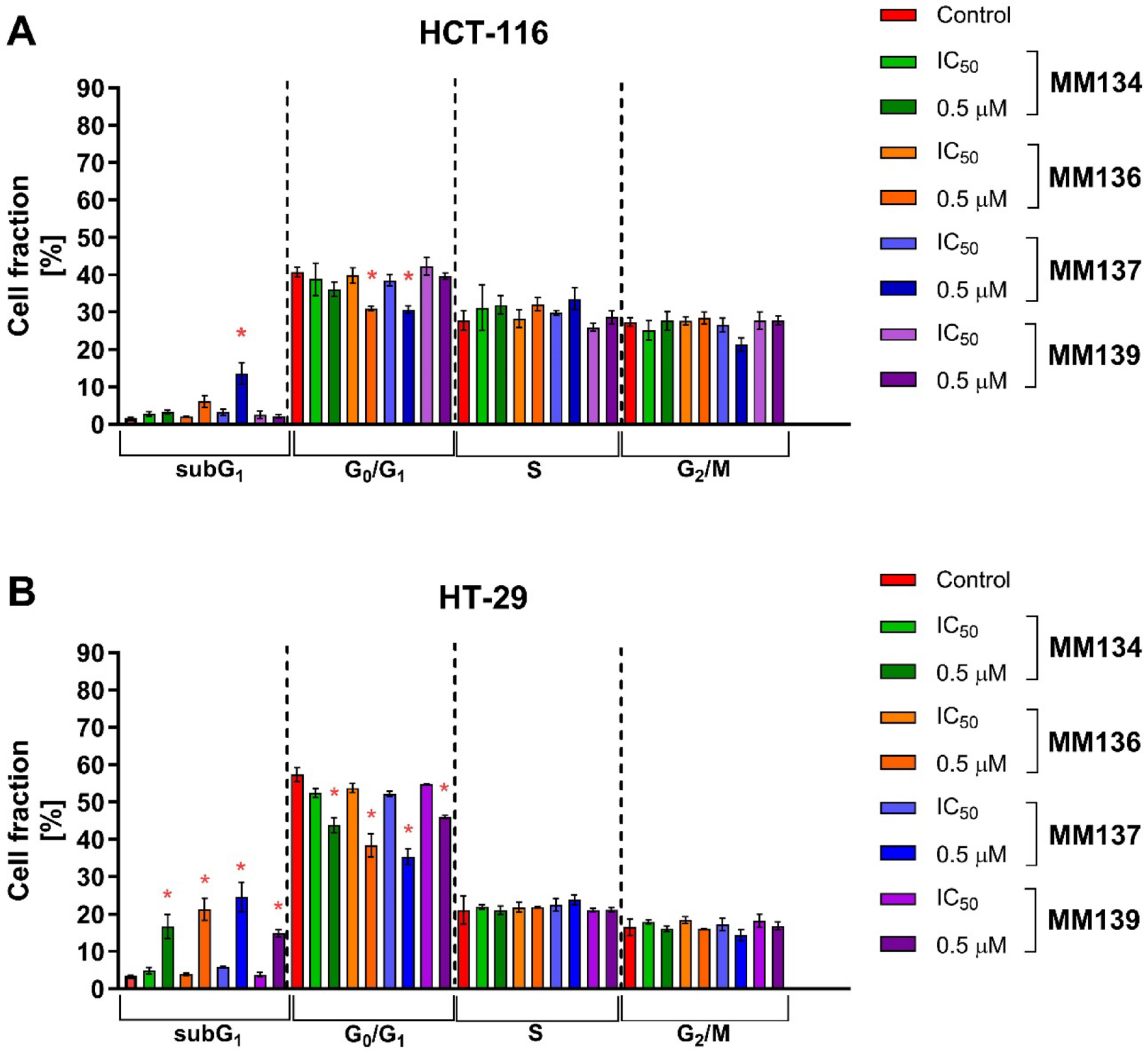
The cell cycle distribution of HCT-116 (**A**) and HT-29 (**B**) cells following 24-h treatment with consensus IC_50_ and 0.5 µM concentrations of **MM134**, **−136**, **−137**, and **− 139** compounds. Data were presented as the mean percentage of cells in the given cell cycle phase (subG_1_, G_0_/G_1_, S, and G_2_) ± SD values. The differences between the experimental samples and control samples were estimated by the ANOVA test followed by post-hoc Dunnett’s test (*p* < 0.05, *N* = 1 × 10^4^).

The distribution of cells in the cell cycle following treatment with **MM**-compounds in HCT-116 and HT-29 cells was shown in Fig. 7A and **B**, respectively.

Statistically significant (*p* < 0.05) increase in the number of apoptotic cells (subG_1_ fraction) was observed following 24-h treatment of HCT-116 with 0.5 µM concentration of **MM137** (subG_1_ cells = 13.6 ± 2.87%; *p* = 0.047) compared with negative control (subG_1_ cells = 1.5 ± 0.35%). At the same time, it induced a decrease in cells in G_0_/G_1_ (G_0_/G_1_ cells = 30.7 ± 0.95%; *p* = 0.0276). Similarly, **MM136** used in the same concentration induced decrease of cells in the G_0_/G_1_ phase (G_0_/G_1_ cells = 30.9 ± 0.6%; *p* = 0.0045). No statistically significant changes were detected for S and G_2_/M phase cells (Fig. 7A).

In contrast, in HT-29 cells all **MM** compounds used in 0.5 µM induced statistically significant increase of sub-G_1_ cells (*p* < 0.05) that were as follows: **MM134** (subG_1_ cells = 16.7 ± 3.15%; *p* = 0.049), **MM136** (subG_1_ cells = 21.3 ± 2.9%; *p* = 0.0281), **MM137** (subG_1_ cells = 24.6 ± 3.9%; *p* = 0.0306), **MM139** (subG_1_ cells = 14.8 ± 0.9%; *p* = 0/0048) compared with negative control (subG_1_ cells = 3.42 ± 0.19%) and a decrease of cells in G_0_/G_1_ phase that that were as follows: **MM134** (G_0_/G_1_ cells = 43.8 ± 2%; *p* = 0.0097) concentration, **MM136** (G_0_/G_1_ cells = 38.3 ± 3.05%; *p* = 0.0335), **MM137** (G_0_/G_1_ cells = 35.4 ± 2.1%; *p* = 0.0009), **MM139** (G_0_/G_1_ cells = 46 ± 0.38%; *p* = 0.0167) compared with negative control (G_0_/G_1_ cells = 57.3 ± 1.86%) (Fig. 7B).

#### Spheroid cell viability

**Fig. 8:**
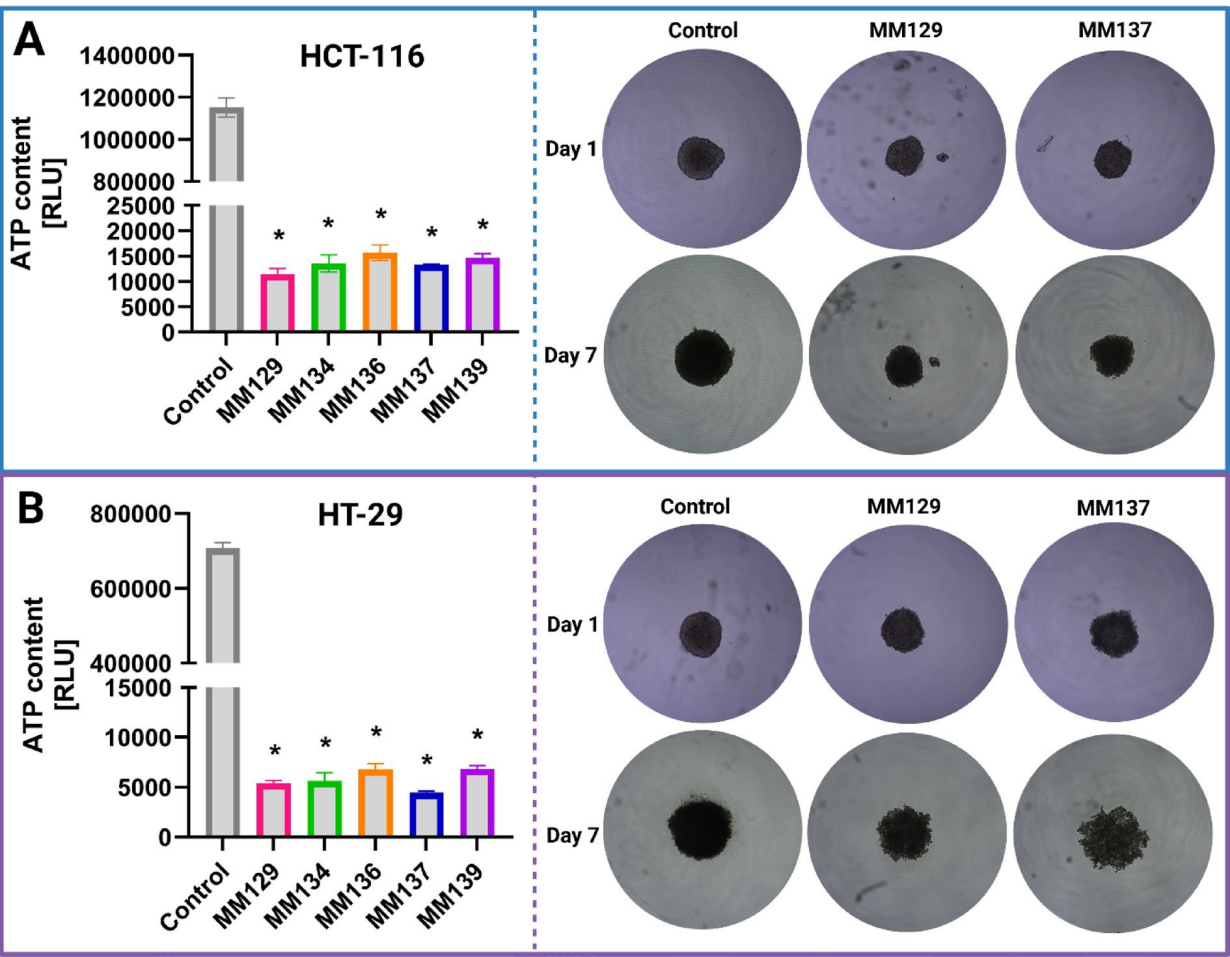
Cell viability of HCT-116 (**A**) and HT-29 (**B**) cells treated with **MM**-compounds (5 µM) in spheroid 3D cell culture using CellTiter-Glo Luminescent Cell Viability Assay. ATP content in spheroid lysates was estimated and provided as luminescence units [RLU] ± SD. The decrease in fluorescence reflects the higher abundance of dead cells in samples. The differences between the experimental and control groups were estimated by the ANOVA test, followed by post-hoc Dunnett’s test (* p = 0.0001, *N* = 4). The examples of images of the control, as well as **MM129** and **MM137**-treated spheroids following 1 day (24 h) and 6 days of incubation, were shown. Created in BioRender. Kciuk, M. (2026) https://BioRender.com/tzk62l5.

Viability of HCT-116 and HT-29 cells treated with **MM**-compounds (5 µM) and grown in a spheroid cell model was incorporated in our study to better reflect the efficacy of the investigated agents (Fig. 8).

**MM-compounds** induced statistically significant (*p* < 0.0001) decrease in ATP content, reflecting loss of cell viability in both cancer cell lines compared to control samples (RLU = 1150852 ± 45198 and RLU = 706769 ± 16082 for HCT-116 and HT-29 cells, respectively. In HCT-116 cells, **MM129** (RLU = 11475 ± 1100) and **MM137** (RLU = 13291 ± 157) triggered the highest reduction in cell viability. Similarly, the highest decrease in ATP content was observed for **MM137** (RLU = 4470 ± 143) and **MM129** (RLU = 5432 ± 264).

### Voltammetric studies

**Fig. 9:**
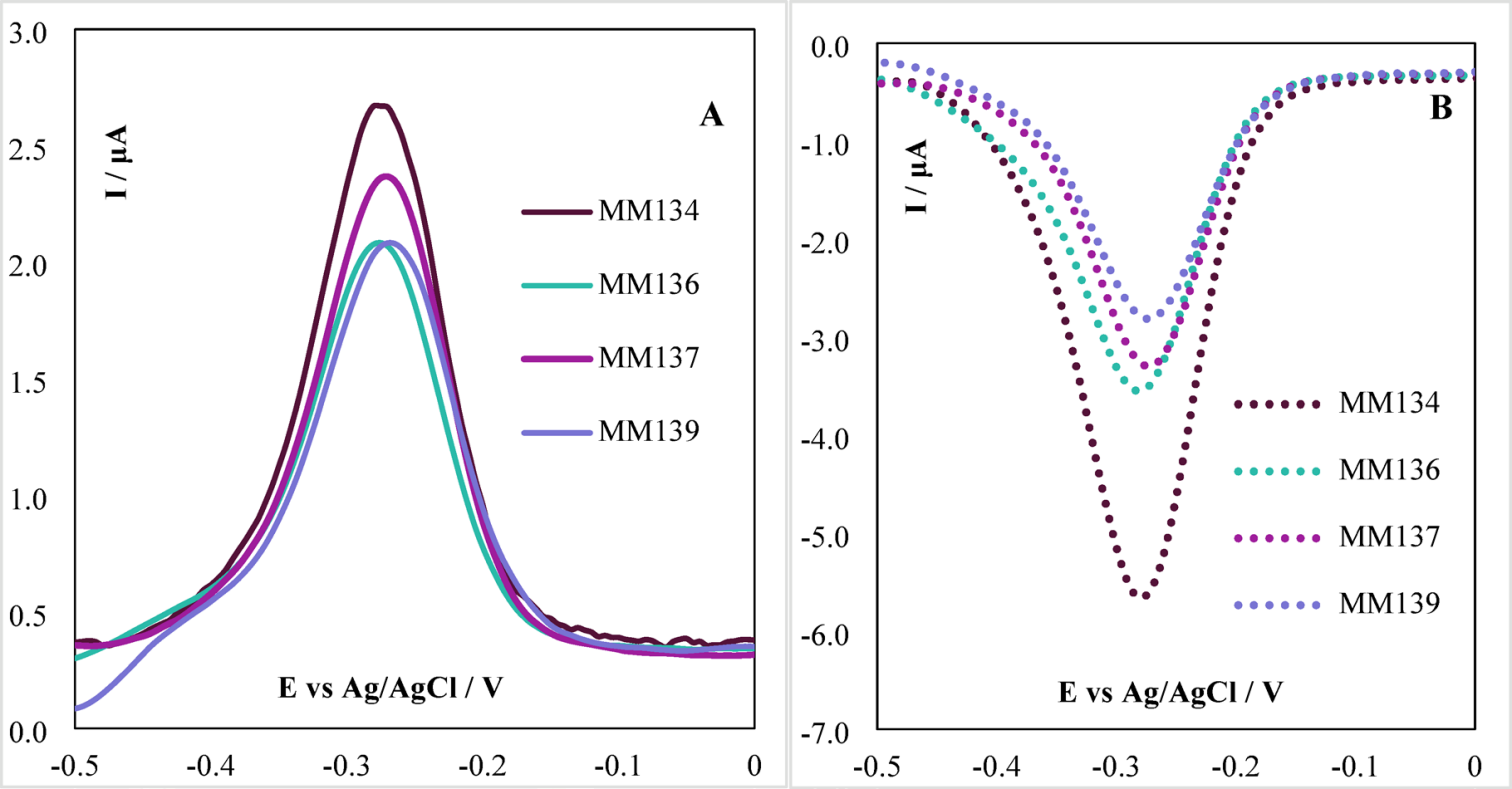
SW voltammograms of **MM134**, **−136**, **−137**, and **− 139**, recorded with anodic (**A**) and cathodic (**B**) direction of the potential sweep, *c* (_**MM**−compounds_) = 1.0 × 10^− 5^ mol L^− 1^; supporting electrolyte: PBS pH 7.4; SW parameters amplitude 25 mV, step potential 5 mV, and frequency of 25 Hz.

The electrochemical behavior of **MM134**, **−136**, **−137**, and **− 139** was first examined with SWV under physiological pH (Fig. 9A-B).

As shown in Fig. 9, in the examined potential window, both single well-shaped oxidation and reduction signals were observed. As can be seen, the highest signals were recorded for **MM134**, regardless of potential sweep direction, while the lowest currents were observed in the case of **MM139** (reduction) or **MM139** and **MM136** (oxidation). All of the tested compounds exhibited similar peak potentials (ca. **−**0.265 V vs. Ag/AgCl for oxidation, and ca. **−**0.275 V vs. Ag/AgCl for reduction processes), suggesting the occurrence of the same electrode mechanisms.

Due to comparable reduction and oxidation peak potentials, the next studies were performed with cyclic voltammetry (data not shown). All tested compounds exhibited one irreversible reduction signal. Those signals were examined using different scan rates, revealing linear dependence between the peak currents (*I*_p_) of **MM134**, **−136**, **−137**, and **− 139** and the square root of the scan rate (*v*^1/2^), suggesting that recorded currents have a diffusional origin^50^.

**Fig. 10:**
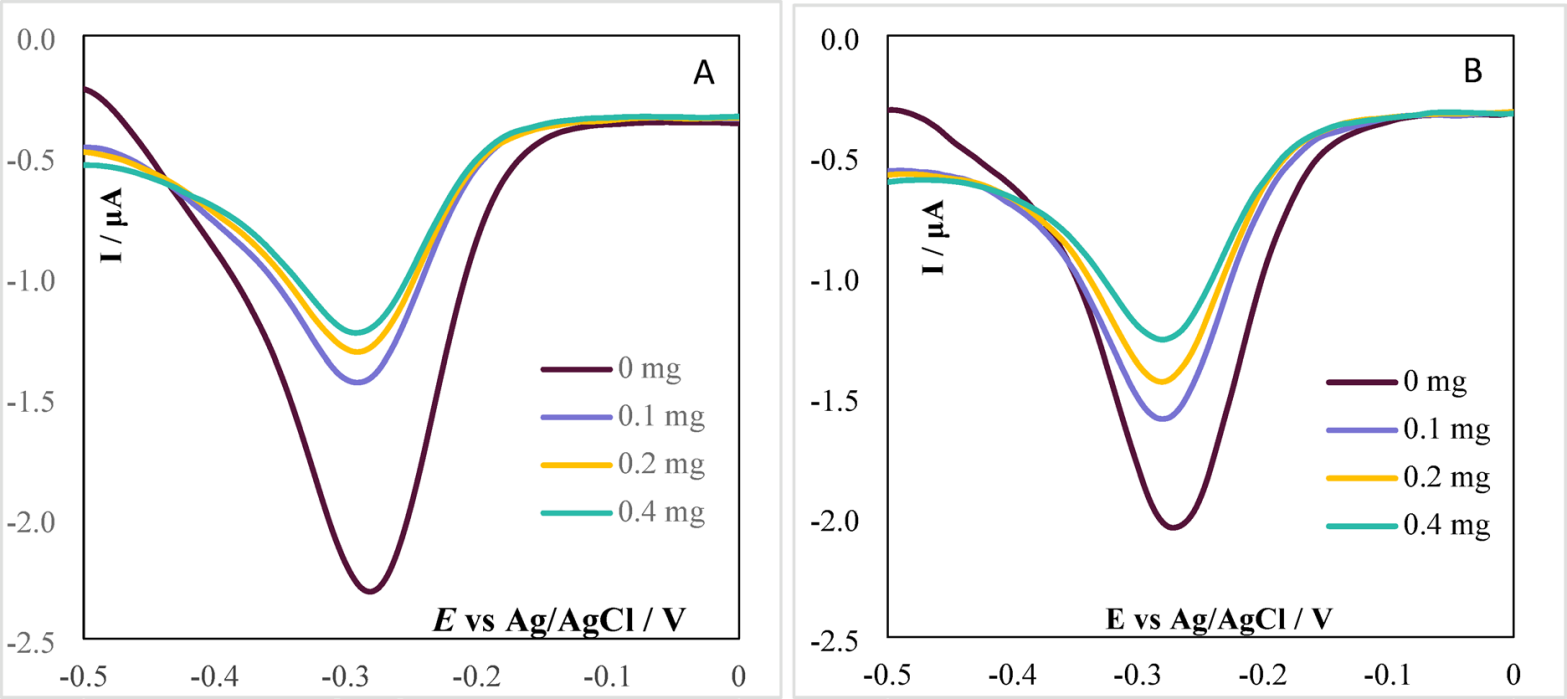
SW voltammograms of **MM136 **(**A**) and **MM139 **(**B**) recorded in the absence and presence of dsDNA (DNA amount from 0 up to 0.4 mg), *c* (_**MM**−compounds_) = 5.0 × 10^− 6^ mol L^− 1^, supporting electrolyte: PBS pH 7.4; SW parameters amplitude 25 mV, step potential 5 mV, and frequency o25 Hz.

In the next step, the interaction of **MM134**, **−136**, **−137**, and **− 139** with dsDNA was investigated using the SWV technique in the potential range from 0 to −0,5 V (in such conditions, dsDNA does not show any electrochemical response). The addition of dsDNA to the sulfonamide solutions significantly decreased the peak currents of all the compounds. Exemplary voltammograms received during the mentioned studies are shown in Fig. 10.

Therefore, it can be concluded that the observed decreases in the peak currents of **MM134**,** −136**,** −137**, and **− 139** in the presence of dsDNA are caused by the binding of the compound to the slowly diffusing large dsDNA molecule, resulting in a significant decrease in the diffusion coefficient value. It is well known that an intercalation-type interaction can be easily revealed when a peak potential shift is observed. As shift potential was not observed in all performed experiments, it can be suggested that different types of interaction occur between **MM134**, **−136**, **−137**, and **− 139** and dsDNA (e.g. groove binding, covalent bonding, etc.).

## Discussion

CRC remains a major global health burden, ranking as the third most common cancer and the second leading cause of cancer-related deaths worldwide^1,51,52^. Chemotherapy constitutes one of the major forms of CRC treatment. It can be used at various stages of CRC, including adjuvant therapy (post-surgery to eliminate remaining cancer cells), neoadjuvant therapy (pre-surgery to shrink tumors), and for advanced or metastatic cancer, where it aims to control disease progression and relieve symptoms. The need for the development of new chemotherapeutic drugs for CRC treatment arises from several significant challenges and limitations associated with current therapies. These include issues related to efficacy, resistance, side effects, and the overall complexity of cancer biology. The heterogeneity of CRC presents a significant obstacle to effective treatment. This heterogeneity manifests in various forms, including genetic, molecular, and cellular diversity within and between tumors^53–55^.

Pyrazolo[4,3-*e*]tetrazolo[1,5-*b*][1,2,4]triazines are characterized by a distinctive fused ring system comprising a pyrazole, tetrazole, and triazine ring. The pyrazole, a five-membered ring containing two adjacent nitrogen atoms^56,57^, combines with the tetrazole, which incorporates four nitrogen atoms in a five-membered structure^58–60^, and the triazine, a six-membered ring with three nitrogen atoms^61,62^. This fusion results in a rigid, planar framework that facilitates a wide range of interactions with biological targets, thereby enhancing their potential as anticancer agents^12^.

We have confirmed that the incorporation of tetrazole in pyrazolo[4,3-*e*][1,2,4]triazine (**MMASZ**) structure into the novel series of compounds (**MM**) induced a significant rise in the cytotoxic activity of the agents (for particular chemical entities above 50-fold). Furthermore, it appeared to increase the cytotoxic selectivity of the compounds towards cancer cells with more clear and stable activity trends (SI = 2.2–3.3 for pyrazolo[4,3-*e*][1,2,4]triazines compared to SI = 3.6–9.6 for pyrazolo[4,3-*e*]tetrazolo[1,5-*b*][1,2,4]triazines). Note that for several **MMASZ** compounds (without tetrazole), we could not obtain reliable selectivity indices due to low or no activity (concentrations > 50 µM) and lack of solubility of the compounds in higher concentrations. In contrast, **MM** compounds were characterized by superior solubility that did not affect the results and provided consistent findings. This could be attributed to several reasons. The tetrazole ring is electron-rich due to its high nitrogen content, leading to strong hydrogen bonding and metal coordination capabilities. It can act as a bioisostere of carboxylic acids and amides, enhancing drug properties like metabolic stability. Compared to carboxyl groups, tetrazole is also more resistant to enzymatic degradation (e.g., amidases, esterases) that favour its use in medicinal chemistry. Additionally, in systems featuring a terminal tetrazole ring, valence tautomerism may occur, resulting in an equilibrium between the azide derivative and a corresponding tricyclic system. This equilibrium is highly dependent on the surrounding environment, including solvent properties, which can modulate the relative stability of different tautomeric forms. Tautomeric equilibrium represents an interesting phenomenon in medicinal chemistry, as distinct tautomers of the same molecule often exhibit varied physicochemical characteristics, including alterations in solubility, lipophilicity, and hydrogen-bonding capacity. These variations can, in turn, significantly impact biological activity, receptor binding, and metabolic stability. Given the profound implications of tautomerism, the rational design of tetrazole-based therapeutics must account for environmental factors that influence tautomeric distribution, ensuring the optimization of pharmacokinetic and pharmacodynamic properties as discussed by other authors^12,58–60,63^.

In our pyrazolo[4,3-*e*][1,2,4]triazine (**MMASZ**) and pyrazolo[4,3-*e*]tetrazolo[1,5-*b*][1,2,4]triazines (**MM**) series, a phenylsulfonyl linker was provided to incorporate various heterocyclic terminal moieties, including methyl 4-hydroxypyrrolidine-2-carboxylate (**MMASZ44** and **MM129**), 2-morpholinoethanamine (**MMASZ25** and **MM134**), morpholine (**MMASZ23** and **MM136**), 1-methylpiperazine (**MMASZ26** and **MM137**), and piperidine (**MMASZ22** and **MM139**). In contrast, **MMASZREF** and **MMREF** compounds were designed to contain amino group. We have, however, not found any clear relationship between the addition of specific moieties and the enhancement of the cytotoxic properties of the compounds.

The interpretation of selectivity indices for the investigated compounds should be considered in the context of the current limitations in available reference standards. To the best of our knowledge, there are no clinically used chemotherapeutic agents or well-established reference compounds that share the same pyrazolo[4,3-*e*]tetrazolo[1,5-*b*][1,2,4]triazine or related scaffold. This structural uniqueness makes direct comparison of SI values with standard anticancer drugs inherently challenging and limits the ability to benchmark the selectivity of the studied derivatives against a well-defined standard within the same chemical class.

Although 5-FU was included as a reference compound, its activity under the present experimental conditions did not allow for a meaningful comparison of selectivity. Specifically, 5-FU did not achieve substantial cytotoxic effects in the tested CRC lines at concentrations up to 50 µM, as evidenced by the absence of pronounced reductions in cell viability (below 50%). Consequently, it was not possible to accurately determine its IC₅₀ values across both cancerous and normal colon epithelial cells, which precludes reliable calculation of its SI in this model.

In contrast, the **MM**-compounds demonstrated measurable cytotoxicity within a lower concentration range, enabling SI determination and revealing notable differences in cancer cell selectivity, particularly for **MM136** and **MM134**. Therefore, while the obtained SI values clearly indicate that specific structural modifications enhance preferential anticancer activity, their direct comparison to standard chemotherapeutics remains limited due to both scaffold dissimilarity and the insufficient responsiveness of the selected cell lines to 5-FU under the applied conditions.

Regardless of the specific moiety added to the compound’s structure, the pyrazolo[4,3-*e*]tetrazolo[1,5-*b*][1,2,4]triazine sulfonamides have shown remarkable cytotoxic activity with IC_50_ concentrations ranging from 0.12 to 1.1 µM. Furthermore, based on the cytotoxicity data and selectivity parameters, we have selected four understudied compounds for further investigation, **MM134**, **−136**, **−137** and **− 139** in CRC cell lines with different genetic and molecular backgrounds (Table S6; Based on^64–67^).

The enhanced activity of **MM** derivatives can be attributed to their multifaceted mechanisms of action. The suppression of cell proliferation, as observed in the BrdU incorporation assay, indicates that these compounds effectively inhibit DNA synthesis, a crucial process for cancer cell growth and division. This inhibition suggests that **MM** derivatives may target cell cycle regulatory pathways, thereby preventing the proliferation of cancer cells^68–70^.

The induction of DNA damage following incubation of cancer cells with the tested compounds was also found utilizing alkaline and neutral comet assay. DNA damage is a critical event that elicits various cellular responses to prevent the propagation of mutations that could lead to oncogenesis. Upon encountering DNA lesions, cells may undergo cell cycle arrest, apoptosis, or senescence, each of which serves as a crucial mechanism for eliminating potentially cancerous cells. Cell cycle arrest, often mediated by the cellular tumor antigen p53 (TP53) tumor suppressor protein, allows time for DNA repair before cell division proceeds. Activation of TP53 leads to the induction of P21, a cyclin-dependent kinase (CDK) inhibitor, which halts the cell cycle in the G_1_ phase. If the damage is irreparable, cells may be directed toward apoptosis or other types of cell death^71–73^. The ability of **MM** derivatives to induce significant DNA damage suggests that they may act as genotoxic agents, directly affecting the genetic material of cancer cells and triggering cell death pathways^74^.

The DNA fragmentation observed in both alkaline and neutral comet assays was further confirmed in the cell cycle analysis of cells following staining their DNA with PI. An increase in the sub-G1 cell fraction indicated the involvement of apoptotic cell death in the cytotoxicity of **MM** compounds. Furthermore, a concurrent decrease in G₀/G₁ phase suggests that cells initially in this phase are undergoing apoptosis rather than progressing to S or G₂/M phases. Nevertheless, apoptotic cell identification should be coupled with other indicative changes, including morphologic alterations, such as nuclear condensation, emergence of PS on the plasma membrane, activation of caspase enzymes, and disruption of mitochondrial membranes^75^.

The electrochemical data provided insight into the mode of interaction between the studied compounds and dsDNA. The observed decrease in peak currents for **MM134**, **−136**, **−137**, and **− 139** in the presence of dsDNA indicates the formation of compound–DNA complexes characterized by reduced diffusion coefficients, consistent with binding to a large, slowly diffusing biomolecule. Importantly, no significant shifts in peak potentials were detected in any of the experiments. Since classical intercalative binding is typically associated with noticeable potential shifts due to strong π–π stacking interactions between the ligand and DNA base pairs, the absence of such changes suggests that intercalation is unlikely to be the dominant mechanism in this case. Instead, the results point toward non-intercalative modes of interaction, such as groove binding or possible external electrostatic associations with the DNA backbone. In conclusion, our findings suggest that the compounds interact with DNA primarily through non-intercalative modes, such as groove binding or electrostatic interactions, which may contribute to their anticancer activity by interfering with DNA-related processes like replication and transcription. Such interactions support DNA binding as a plausible mechanism of action underlying their cytotoxic effects.

IInterestingly, studies of other groups on different pyrazolo[4,3-*e*]tetrazolo[1,5-*b*][1,2,4]triazine sulfonamide derivatives indicated apoptosis as the main mechanism of action of this class of compounds^23,28,76–78^. In contrast, our studies indicated that other cell death pathways, such as necroptosis, may also be involved^20^, as we have not observed DNA fragmentation in the flow cytometry experiments, DNA laddering, and apoptotic gene expression changes in cancer cells subjected to the activity of the **MM**-compounds. Despite that, we have detected other hallmarks of apoptosis, e.g., PS exposition, morphological features, and MMP changes in cells subjected to tested derivatives^20,27,36^. This may suggest a differential response of cancer cells (of different origins) to the tested compounds.

Surprisingly, we found that **MM137** and **MM134** exhibit necrotic effects at early time points, particularly in HCT-116 and HT-29 cells, respectively, while **MM136** demonstrates a time-dependent reduction in necrosis, supporting its dominant apoptotic mode of action. This observation could be attributed to the fact that during the early phase of treatment (24 h), cells may experience acute stress from the compounds, leading to rapid loss of membrane integrity, which is characteristic of necrosis. This can occur when the cellular damage exceeds the immediate capacity of the apoptotic machinery to activate controlled cell death pathways. Necrosis is often a default result when cells experience severe metabolic stress, oxidative damage, or energy depletion in the initial response to a cytotoxic compound. Apoptosis is a highly regulated and time-dependent process that involves the sequential activation of signaling cascades (e.g., caspases), chromatin condensation, and membrane blebbing. It takes time for the apoptotic machinery to fully engage and complete the process of programmed cell death^79,80^. At 24 h, many cells may still be in the early stages of apoptosis, but the markers of this cell death type are not yet visible under certain detection methods. As a result, these cells may be misinterpreted as necrotic. Some cells that initially undergo necrosis-like changes at 24 h may be reclassified as apoptotic at later time points. This is because secondary necrosis can occur when apoptotic cells are not cleared efficiently by phagocytosis, resulting in the breakdown of their membrane integrity, resembling necrosis. At 48 h, these events are more distinguishable. The methods used to distinguish apoptotic and necrotic cells (e.g., AO/EB, HE/JP staining) might overestimate necrotic cells at 24 h due to early loss of membrane integrity or incomplete apoptosis. At 48 h, apoptosis becomes more apparent as the cells complete the regulated death process^81,82^.

To comprehensively evaluate the anticancer potential of pyrazole-tetrazole-sulfonamides, we employed a 3D spheroid model, along with CellTiter-Glo to assess their cytotoxic effect. The spheroid model was chosen to better recapitulate the tumor microenvironment, mimicking key features of solid tumors such as hypoxia, cell-cell interactions, and drug penetration barriers, thereby providing a more physiologically relevant assessment of compound activity compared to traditional 2D cultures. The combined use of these models enhances the translational relevance of our findings, facilitating the rational development of pyrazole-tetrazole-sulfonamides as promising anticancer agents. Similar to 2D cell cultures, **MM129** and **MM137** were indicated as the most potent inducers of cell death in both HCT-116 and HT-29 cells. Both compounds were effective in conenctrations as low as 5 µM.

These results indicate pyrazole-triazine compounds as potential anticancer agents. Nevertheless, preclinical in vivo (animal) studies are a critical step in drug design and provide key insights into the efficacy of a drug candidate by demonstrating its therapeutic effects within a living organism. Additionally, in vivo studies enable the evaluation of pharmacokinetics and pharmacodynamics, offering detailed information on how the drug is absorbed (A), distributed (D), metabolized (M), and excreted (E), as well as its mechanism of action and the relationship between drug concentration and biological effects. Safety and toxicology (T) assessments in animal models are crucial for identifying potential adverse effects, determining the therapeutic window, and establishing safe dosing regimens, all of which are prerequisites for advancing to human trials^83–85^. Promising results were published in 2021 by Hermanowicz et al^21^., where the **MM129** derivative exhibited rapid absorption with a bioavailability of 68.6% following intraperitoneal administration. Importantly, no serious adverse events were associated with **MM129** administration, indicating a favorable safety profile. The compound was not lethal or toxic at an anticancer-effective dose of 10 µmol/kg in mice. Furthermore, after 14 days of treatment, hematological and biochemical parameters, as well as liver and renal functions, remained within normal ranges, further supporting its safety. Similarly, zebrafish embryos treated with a 10 µM concentration of **MM129** did not exhibit any sublethal effects. These findings suggest that **MM129** and other **MM**-compounds could constitute potentially safe and well-tolerated anticancer agents, offering promise for future clinical use in patients with colon cancer^21^. Accordingly, our in silico investigations suggested that **MM134**, **−136**, **−137**, and **− 139** may exhibit better ADMET properties than previous derivatives. Future studies will focus on elucidating the detailed molecular mechanisms underlying the anticancer effects of **MM** derivatives, optimizing their pharmacokinetic and pharmacodynamic profiles, and assessing their efficacy and safety in in vivo models^27^.

Several studies indicated a possible mechanism of action of a class of **MM** compounds that is multi-targeted and involves the modulation of key signaling pathways responsible for tumor cell survival, proliferation, and immune evasion. In particular, **MM129** has been identified as an inhibitor of the Bruton’s tyrosine kinase (BTK)/phosphoinositide 3-kinase (PI3K)/AKT serine/threonine kinase (AKT)/mammalian target of rapamycin (mTOR) axis, leading to suppression of pro-survival signaling and induction of cell cycle arrest at the G_0_/G_1_ phase^19,21^. The downregulation of AKT and mTOR pathways is especially relevant, as these kinases are central regulators of cancer cell metabolism, growth, and resistance to therapy. Moreover, **MM129** was shown to decrease programmed death-ligand 1 (PD-L1) expression, suggesting its potential role in overcoming tumor immune escape mechanisms by restoring anti-tumor immune responses^19^.

Another important aspect of **MM** compounds’ activity is their impact on oxidative stress and inflammatory signaling. The pro-oxidative properties, manifested by increased reactive oxygen species (ROS) levels, can enhance cellular damage and sensitize cancer cells to apoptosis^20^. Concurrently, modulation of nuclear factor kappa-light-chain-enhancer of activated B cells (NF-κB) signaling and cytokine expression suggests that these compounds may interfere with inflammation-driven tumor progression. Furthermore, recent findings demonstrated that **MM129** can regulate the sirtuin 1 (SIRT1)/signal transducer and activator of transcription 3 (STAT3) signaling pathway, leading to suppression of senescence-associated secretory phenotype (SASP) factors such as interleukin 6 (IL-6) and tumor necrosis factor alpha (TNF-α), thereby limiting pro-tumorigenic inflammation and reversing therapy-induced senescence^26,86^.

Importantly, **MM** compounds also influence autophagy and metabolic adaptation of cancer cells. While some derivatives inhibit autophagy-related proteins, others may induce autophagic processes depending on the cellular context, often through mTOR inhibition, highlighting a complex interplay between autophagy and apoptosis^87^. This dual role may contribute to overcoming chemoresistance, particularly when **MM129** is used in combination therapies. Indeed, synergistic effects have been observed with agents such as 5-FU and indoximod, resulting in enhanced apoptosis, disruption of mitochondrial function, and inhibition of additional targets such as indoleamine 2,3-dioxygenase 1^77^.

The study highlights pyrazolo[4,3-*e*]tetrazolo[1,5-*b*][1,2,4]triazine sulfonamides as promising anticancer candidates with potent cytotoxicity, favorable selectivity, and potential multimodal mechanisms involving apoptosis and necroptosis. Preliminary in vivo data from previous work confirm favorable pharmacokinetic and safety profiles of related derivatives. These findings establish a foundation for further preclinical evaluation of **MM** compounds as prospective therapeutic agents for CRC.

## Conclusions

CRC continues to pose a major clinical challenge due to late diagnosis, treatment resistance, and adverse side effects associated with current chemotherapeutics. The present study demonstrates that structural modification of pyrazolo[4,3-*e*][1,2,4]triazine scaffolds by introducing tetrazole functionalities results in a new class of potent and selective anticancer agents.

The incorporation of the tetrazole moiety significantly enhanced cytotoxicity, solubility, and selectivity, likely through improved hydrogen bonding and bioisosteric properties. The **MM** derivatives displayed submicromolar cytotoxic effects across CRC cell lines, induced DNA damage, inhibited DNA synthesis, and triggered both apoptotic and necroptotic pathways, depending on cellular context and exposure duration. Electrochemical and 3D spheroid experiments further confirmed strong compound–DNA interactions and biological relevance within a tumor-like microenvironment.

Among the tested derivatives, **MM136** and **MM137** exhibited the most promising activity profiles, combining potent cytotoxic effects with favorable selectivity indices. Existing in vivo data on **MM129** also support the safety and bioavailability of this compound class, underscoring their translational potential.

## Supplementary Information

Below is the link to the electronic supplementary material.

Supplementary Material 1

## Data Availability

The data presented in this study are available in the main text of this article/supplementary materials of this article or on request from the corresponding author.

## Abbreviations

5-FU: 5–fluorouracil
ADMET: Absorption, distribution, metabolism, excretion, and toxicity
AKT: AKT serine/threonine kinase
AO: Acridine orange
APC: Adenomatous polyposis coli
ATCC: American type culture collection
BCL: 2–B–cell lymphoma 2
BrdU: 5–bromo–2′–deoxyuridine
BSA: Bovine serum albumin
BTK: Bruton’s tyrosine kinase
CDK: Cyclin–dependent kinase
CIMP: CpG island methylator phenotype
CRC: Colorectal cancer
CV: Cyclic voltammetry
DAPI: 4′,6–diamidino–2–phenylindole
DMSO: Dimethyl sulfoxide
DSBs: DNA double–strand breaks
dsDNA: Double–stranded DNA
EB: Ethidium bromide
EDTA: Ethylenediaminetetraacetic acid
FAP: Familial adenomatous polyposis
FBS: Fetal bovine serum
FITC: Fluorescein isothiocyanate
HDAC: Histone deacetylase
HE: Hoechst 33342
HRMS: High–resolution mass spectrometry
IC_50_: 50% inhibitory concentration
IL: 6–interleukin 6
KRAS: kirsten rat sarcoma viral oncogene homolog
LMP: Low melting point agarose
mTOR: Mammalian target of rapamycin
MTT: (4,5–dimethylthiazol–2–yl)–2,3–diphenyltetrazolium bromide
NF: κB–nuclear factor kappa–light–chain–enhancer of activated B cells
NMP: Normal melting point agarose
NMR: Nuclear magnetic resonance
OXA: Oxaliplatin
PBS: Buffered saline
PD: L1–programmed death–ligand 1
PFA: Paraformaldehyde
PI: Propidium iodide
PI3K: Phosphoinositide 3–kinase
PIK3CA: Phosphatidylinositol–4,5–bisphosphate 3–kinase catalytic subunit alpha
PTEN: Phosphatase and tensin homolog
RLU: Relative luminescence units
ROS: Reactive oxygen species
SASP: Senescence–associated secretory phenotype
SIRT1: Sirtuin 1
STAT3: Signal transducer and activator of transcription 3
SD: Standard deviation
SI: Selectivity index
SN: 38–7–ethyl–10–hydroxycamptothecin
SSBs: DNA single–strand breaks
SWV: Square–wave voltammetry
TLC: Thin–layer chromatography
TNF: α–tumor necrosis factor alpha
TP53: Cellular tumor antigen p53
TRIS: Tris(hydroxymethyl)aminomethane
ULAP: Ultra-low attachment plate
