# Supplementary material for: Targeting colorectal cancer with pyrazolo[4,3-e][1,2,4]triazine and pyrazolo[4,3-e]tetrazolo[1,5-b][1,2,4]triazine sulfonamides: comprehensive in vitro evaluation
Source: Sci Rep. 2026 May 31;16:24889. doi: 10.1038/s41598-026-52451-5 (PMC13458726; doi:10.1038/s41598-026-52451-5)
Supplement: Supplementary file 1 [file 41598_2026_52451_MOESM1_ESM.doc]

### Supplementary Information

**Targeting colorectal cancer with pyrazolo[4,3-*e*][1,2,4]triazine and pyrazolo[4,3-*e*]tetrazolo[1,5-*b*][1,2,4]triazine sulfonamides: comprehensive *in vitro* evaluation**

**Mateusz Kciuk^1,2^*, Gabriela Machura^3,4^, Katarzyna Wanke^1,5^, Sylwia Smarzewska^4^, Mariusz Mojzych^6^, Elżbieta Płuciennik^2^ and Renata Kontek^1^**

^1^ University of Lodz, Department of Molecular Biotechnology and Genetics, Banacha 12/16, 90-237 Lodz, Poland.

^2^ Medical University of Lodz, Faculty of Medicine, Department of Functional Genomics, Zeligowskiego 7/9, 90-752 Lodz, Poland.

^3^ BioMedChem Doctoral School of the University of Lodz and Lodz Institutes of the Polish Academy of Sciences, Lodz, Poland.

^4^ University of Lodz, Department of Inorganic and Analytical Chemistry, 12 Tamka Str, 91-403 Lodz, Poland.

^5^ University of Lodz, Doctoral School of Exact and Natural Sciences, Banacha Street 12/16, 90-237 Lodz, Poland.

^6^ The Mazovian Academy in Płock, Collegium Medicum, Plac Dąbrowskiego 2, 09-402 Płock, Poland.

* Correspondence: mateusz.kciuk@biol.uni.lodz.pl

**Table S1.** Results of 24-h crystal violet assay. IC_50_ values from two independent experiments (IC_50(1)_ and IC_50(2)_) with corresponding coefficients of determination (R^2^), 95% confidence interval (95%CI) values and calculated mean IC_50_ values ± SD [μM] were provided.

| **MM compound** | **IC_50(1)_** | **R^2^_1_** | **95%CI of IC_50_** | **IC_50(2)_** | **R^2^_2_** | **95%CI of IC_50_** | **Mean IC_50_ ± SD** |
| --- | --- | --- | --- | --- | --- | --- | --- |
| **DLD-1 cell line** | | | | | | | |
| MMASZREF | 12.9 | 0.94 | 9.19–17.3 | 13.16 | 0.95 | 9.77–17.3 | 13.03 ± 0.18 |
| MMREF | 0.8 | 0.98 | 0.72–0.89 | 0.9 | 0.99 | 0.87–0.95 | 0.85 ± 0.07 |
| MMASZ44 | 18.3 | 0.9 | 12.9–25.2 | 17.7 | 0.9 | 12.08–25.8 | 18 ± 0.42 |
| MM129 | 0.99 | 0.97 | 0.83–1.16 | 1.095 | 0.98 | 0.99–1.19 | 1.04 ± 0.07 |
| MMASZ25 | 1.46 | 0.91 | 0.94 – 2.13 | 3.78 | 0.95 | 2.53–5.83 | 2.62 ± 1.64 |
| MM134 | 0.62 | 0.9 | 0.39–0.84 | 0.67 | 0.94 | 0.47–0.83 | 0.64 ± 0.03 |
| MMASZ23 | 11.12 | 0.91 | 7.25–16.2 | 10.8 | 0.94 | 7.18–14.77 | 10.96 ± 0.22 |
| MM136 | 0.94 | 0.96 | 0.77–1.11 | 0.85 | 0.97 | 0.7–0.99 | 0.9 ± 0.06 |
| MMASZ26 | 6.2 | 0.96 | 4.54–8.3 | 6 | 0.95 | 4.34–8.2 | 6.1 ± 0.14 |
| MM137 | 0.3 | 0.99 | 0.29–0.33 | 0.31 | 0.98 | 0.27–0.37 | 0.3 ± 0.007 |
| MMASZ139 _22_ | 1.94 | 0.96 | 1.44–2.61 | 3.66 | 0.93 | 2.67–4.49 | 2.8 ± 1.22 |
| MM139 | 0.32 | 0.95 | 0.2–0.46 | 0.22 | 0.98 | 0.17–0.3 | 0.27 ± 0.07 |
| **HCT-116 cell line** | | | | | | | |
| MMASZREF | 16.9 | 0.93 | 12.36–22.32 | 12.34 | 0.93 | 8.8–17.04 | 14.6 ± 3.22 |
| MMREF | 0.36 | 0.99 | 0.3–0.42 | 0.4 | 0.99 | 0.36–0.44 | 0.38 ± 0.03 |
| MMASZ44 | 14.85 | 0.97 | 12.04–18.23 | 15.51 | 0.96 | 12.3–19.4 | 15.18 ± 0.5 |
| MM129 | 0.47 | 0.95 | 0.36–0.59 | 0.44 | 0.99 | 0.39–0.49 | 0.46 ± 0.02 |
| MMASZ25 | 6.45 | 0.96 | 4.96–8.4 | 4.64 | 0.93 | 2.44–7.28 | 5.54 ± 1.28 |
| MM134 | 0.43 | 0.97 | 0.35–0.52 | 0.34 | 0.99 | 0.3–0.39 | 0.38 ± 0.06 |
| MMASZ23 | 3.97 | 0.98 | 3.04–5.25 | 3.5 | 0.98 | 2.13–5.83 | 3.73 ± 0.33 |
| MM136 | 0.65 | 0.98 | 0.57–0.74 | 0.73 | 0.99 | 0.64–0.82 | 0.69 ± 0.06 |
| MMASZ26 | 2.04 | 0.99 | 1.81–2.29 | 4.99 | 0.99 | 4.3–5.79 | 3.51 ± 2.09 |
| MM137 | 0.23 | 0.95 | 0.16–0.33 | 0.26 | 0.99 | 0.22–0.3 | 0.24 ± 0.02 |
| MMASZ22 | 1.74 | 0.95 | 1.34–2.2 | 1.9 | 0.98 | 1.6–2.25 | 1.82 ± 0.11 |
| MM139 | 0.21 | 0.98 | 0.16–0.27 | 0.18 | 0.99 | 0.16–0.2 | 0.19 ± 0.02 |
| **HT-29 cell line** | | | | | | | |
| MMASZREF | >50 | n/d | n/d | >50 | n/d | n/d | >50 |
| MMREF | 0.78 | 0.98 | 0.67–0.91 | 0.79 | 0.97 | 0.62–0.98 | 0.8 ± 0.007 |
| MMASZ44 | >50 | n/d | n/d | >50 | n/d | n/d | >50 |
| MM129 | 0.88 | 0.98 | 0.73–1.05 | 0.81 | 0.96 | 0.62–1.07 | 0.85 ± 0.05 |
| MMASZ25 | 5.75 | 0.95 | 3.83–8.13 | 8.88 | 0.99 | 7.8–9.9 | 7.31 ± 2.21 |
| MM134 | 0.47 | 0.97 | 0.37–0.59 | 0.57 | 0.98 | 0.46–0.69 | 0.52 ± 0.07 |
| MMASZ23 | 24.3 | 0.7 | 12.55–38.7 | 26.3 | 0.95 | 22.3–30.09 | 25.3 ± 1.4 |
| MM136 | 0.12 | 0.83 | 0.04–0.28 | 0.1 | 0.79 | 0.03–0.24 | 0.11 ± 0.01 |
| MMASZ26 | 8.3 | 0.98 | 7.14–9.5 | 8.6 | 0.9 | 6.35–11.5 | 8.45 ± 0.21 |
| MM137 | 0.2 | 0.96 | 0.15–0.28 | 0.22 | 0.95 | 0.16–0.32 | 0.21 ± 0.014 |
| MMASZ22 | 6.12 | 0.99 | 5.7–6.55 | 3.28 | 0.98 | 2.75–3.9 | 4.7 ± 2 |
| MM139 | 0.19 | 0.9 | 0.11–0.31 | 0.46 | 0.97 | 0.34–0.6 | 0.32 ± 0.19 |
| **CCD-841 CoN cell line** | | | | | | | |
| MMASZREF | >50 | n/d | n/d | >50 | n/d | n/d | >50 |
| MMREF | 2.71 | 0.85 | 1.81–4.3 | 2.84 | 0.96 | 2.28–3.56 | 2.78 ± 0.09 |
| MMASZ44 | 17.7 | 0.92 | 11.8–24.8 | 20.9 | 0.95 | 15.7–26.4 | 19.3 ± 2.26 |
| MM129 | 2.78 | 0.98 | 2.37–3.3 | 2.8 | 0.92 | 2.04–3.9 | 2.79 ± 0.014 |
| MMASZ25 | 10.7 | 0.99 | 9.88–11.9 | 14.7 | 0.94 | 9.66–19.17 | 12.7 ± 2.83 |
| MM134 | 4.9 | 0.97 | 3.8–6.3 | 5.6 | 0.99 | 5.09–6.11 | 5.25 ± 0.49 |
| MMASZ23 | 39.72 | 0.96 | 33.6–46.8 | 29.01 | 0.96 | 23.7–34.7 | 34.4 ± 7.6 |
| MM136 | 5.37 | 0.99 | 4.91–5.86 | 4.77 | 0.98 | 4.08–5.54 | 5.07 ± 0.42 |
| MMASZ26 | 12.05 | 0.98 | 10.46–13.9 | 10.84 | 0.99 | 9.9–12.1 | 11.45 ± 0.86 |
| MM137 | 1.58 | 0.97 | 1.36–1.88 | 1.46 | 0.98 | 1.38–1.67 | 1.52 ± 0.08 |
| MMASZ22 | 8.9 | 0.99 | 8.52–9.25 | 9.58 | 0.99 | 9.17–10.18 | 9.24 ± 0.48 |
| MM139 | 2.19 | 0.97 | 1.44–3.07 | 3.04 | 0.99 | 2.87–3.22 | 2.61 ± 0.6 |

**Table S2.** Results of 24-h MTT assay. IC_50_ values from two independent experiments (IC_50(1)_ and IC_50(2)_) with corresponding coefficients of determination (R^2^), 95% confidence interval (95%CI) values and calculated mean IC_50_ values ± SD [μM] were provided.

| **MM compound** | **IC_50(1)_** | **R^2^_1_** | **95%CI of IC_50_** | **IC_50(2)_** | **R^2^_2_** | **95%CI of IC_50_** | **Mean IC_50_ ± SD** |
| --- | --- | --- | --- | --- | --- | --- | --- |
| **DLD-1** | | | | | | | |
| MMASZREF | 6.92 | 0.96 | 5.15–9.25 | 5.7 | 0.95 | 4–7.9 | 6.3 ± 0.9 |
| MMREF | 0.4 | 0.97 | 0.27–0.58 | 0.77 | 0.95 | 0.52–1.09 | 0.58 ± 0.26 |
| MMASZ44 | >30 | n/d | n/d | >30 | n/d | n/d | >30 |
| MM129 | 1.09 | 0.97 | 0.94–1.27 | 1.18 | 0.96 | 0.95–1.4 | 1.13 ± 0.06 |
| MMASZ25 | 2.02 | 0.92 | 1.21–3.07 | 3.05 | 0.96 | 2.2–4.13 | 2.52 ± 0.71 |
| MM134 | 0.79 | 0.92 | 0.53–1.02 | 0.99 | 0.97 | 0.87–1.12 | 0.89 ± 0.14 |
| MMASZ23 | 14.8 | 0.91 | 9.93–20.7 | 13.5 | 0.88 | 8.45–20.44 | 14.15 ± 0.91 |
| MM136 | 0.43 | 0.94 | 0.29–0.61 | 0.41 | 0.92 | 0.25–0.63 | 0.42 ± 0.01 |
| MMASZ26 | 11.4 | 0.98 | 10.2–12.9 | 12.03 | 0.99 | 10.6–13.6 | 11.7 ± 0.44 |
| MM137 | 0.28 | 0.97 | 0.20–0.36 | 0.29 | 0.94 | 0.18–0.42 | 0.28 ± 0.007 |
| MMASZ22 | 2.52 | 0.93 | 1.59–3.88 | 1.41 | 0.87 | 0.74–2.27 | 1.96 ± 0.78 |
| MM139 | 0.85 | 0.96 | 0.69–1 | 0.9 | 0.97 | 0.77–1.04 | 0.87 ± 0.03 |
| **HCT-116 cell line** | | | | | | | |
| MMASZREF | 3.35 | 0.92 | 1.99–5.17 | 3.23 | 0.92 | 2.03–5.09 | 3.29 ± 0.08 |
| MMREF | 0.36 | 0.99 | 0.3–0.42 | 0.3 | 0.99 | 0.27–0.34 | 0.33 ± 0.04 |
| MMASZ44 | 8.96 | 0.95 | 6.15–12.13 | 12.12 | 0.93 | 7.17–17.44 | 10.54 ± 2.23 |
| MM129 | 0.13 | 0.97 | 0.1–0.17 | 0.1 | 0.98 | 0.08–0.13 | 0.11 ± 0.02 |
| MMASZ25 | 2.08 | 0.96 | 1.49–2.86 | 4.5 | 0.92 | 2.76–6.48 | 3.29 ± 1.71 |
| MM134 | 0.33 | 0.98 | 0.27–0.4 | 0.44 | 0.99 | 0.38–0.5 | 0.38 ± 0.08 |
| MMASZ23 | 9.08 | 0.9 | 5.5–14,38 | 10.97 | 0.99 | 9.7–12.5 | 10.03 ± 1.3 |
| MM136 | 0.25 | 0.97 | 0.18–0.33 | 0.34 | 0.99 | 0.29–0.38 | 0.29 ± 0.06 |
| MMASZ26 | 4.96 | 0.9 | 3.03–7.76 | 1.57 | 0.93 | 0.9–2.44 | 3.36 ± 2.39 |
| MM137 | 0.14 | 0.99 | 0.13–0.15 | 0.15 | 0.99 | 0.13–0.17 | 0.14 ± 0.007 |
| MMASZ22 | 4.06 | 0.98 | 3.75–4.48 | 2.89 | 0.95 | 2.27–3.55 | 3.48 ± 0.83 |
| MM139 | 0.36 | 0.98 | 0.28–0.44 | 0.21 | 0.93 | 0.13–0.33 | 0.28 ± 0.1 |
| **HT-29 cell line** | | | | | | | |
| MMASZREF | >50 | n/d | >50 | >50 | n/d | n/d | >50 |
| MMREF | 0.77 | 0.99 | 0.65–0.89 | 0.71 | 0.96 | 0.52–0.92 | 0.74 ± 0.04 |
| MMASZ44 | >50 | n/d | n/d | >50 | n/d | n/d | >50 |
| MM129 | 0.14 | 0.91 | 0.09–0.22 | 0.16 | 0.97 | 0.13-0.2 | 0.15 ± 0.014 |
| MMASZ25 | 2.86 | 0.96 | 2.53–3.22 | 4.24 | 0.97 | 3.74–4.82 | 3.55 ± 0.98 |
| MM134 | 0.18 | 0.99 | 0.16–0.2 | 0.26 | 0.99 | 0.22–0.3 | 0.22 ± 0.06 |
| MMASZ23 | >30 | n/d | n/d | >30 | n/d | n/d | >30 |
| MM136 | 0.14 | 0.98 | 0.12–0.18 | 0.13 | 0.96 | 0.1–0.18 | 0.13 ± 0.007 |
| MMASZ26 | 2.88 | 0.96 | 2.65–3.16 | 3.67 | 0.89 | 2.79–4.85 | 3.28 ± 0.56 |
| MM137 | 0.12 | 0.98 | 0.09–0.14 | 0.12 | 0.99 | 0.1–0.15 | 0.12 |
| MMASZ22 | 1.63 | 0.96 | 1.39–1.9 | 2.53 | 0.98 | 2.29–2.8 | 2.08 ± 0.64 |
| MM139 | 0.2 | 0.99 | 0.17–0.23 | 0.14 | 0.98 | 0.11–0.17 | 0.17 ± 0.04 |
| **CCD-841 CoN cell line** | | | | | | | |
| MMASZREF | >50 | n/d | n/d | >50 | n/d | n/d | >50 |
| MMREF | 1.94 | 0.96 | 1.5–2.6 | 1.45 | 0.93 | 0.99–2.15 | 1.69 ± 0.35 |
| MMASZ44 | >50 | n/d | n/d | >50 | n/d | n/d | >50 |
| MM129 | 2.34 | 0.98 | 1.89–2.88 | 2.02 | 0.94 | 1.5–2.9 | 2.18 ± 0.23 |
| MMASZ25 | 18.38 | 0.85 | 10.04–28.75 | 10.94 | 0.76 | 4.06–24.97 | 14.66 ± 5.26 |
| MM134 | 2.29 | 0.97 | 1.96–2.72 | 2.67 | 0.97 | 2.25–3.19 | 2.48 ± 0.27 |
| MMASZ23 | >50 | n/d | n/d | >50 | n/d | n/d | >50 |
| MM136 | 2.8 | 0.99 | 2.55–3.08 | 2.5 | 0.98 | 2.21–2.81 | 2.65 ± 0.21 |
| MMASZ26 | 14.9 | 0.98 | 12.6–17.7 | 18.7 | 0.98 | 16.04–21.6 | 16.8 ± 2.69 |
| MM137 | 1.15 | 0.98 | 1.07–1.24 | 1.18 | 0.99 | 1.13–1.27 | 1.16 ± 0.02 |
| MMASZ22 | 3.02 | 0.95 | 2.06–4.34 | 3.48 | 0.96 | 2.51–4.74 | 3.25 ± 0.33 |
| MM139 | 1.28 | 0.99 | 1.19–1.38 | 1.9 | 0.99 | 1.77–2.04 | 1.59 ± 0.44 |

**Figure S1. MTT assay results:** Viability of cancer ((A)DLD-1, (B) HCT-116, (C) HT-29) and normal cells (D) CCD-841 CoN) following 24-h treatment with 5-fluorouracil (5-FU) used in concentration range of 50-400 µM. Data were presented as mean [%] ± SD cell viability. Ordinary one-way ANOVA followed by post-hoc Dunnett's multiple comparisons test were used to estimated statistically significant (*p* < 0.05) differences in cell viability. Experiment included six technical replicated and two biological replicates (*N* = 2; **p* < 0.05; ***p* < 0.01; *** *p* < 0.001; **** *p* < 0.001).

**Figure S2. MTT assay results:** Dose-response curves obtained for **MM134**, **-136**, **-137**, and -**139** compounds in HCT-116 colorectal cancer cells in a 24-h incubation experiment performed in duplicate. The IC_50_ value is the concentration of a tested substance that causes a 50% decrease in cell viability relative to the negative control, which is considered 100% (*N*= 2).

**Figure S3. MTT assay results:** Dose-response curves obtained for **MM134**, **-136**, **-137**, and -**139** compounds in HT-29 colorectal cancer cells in a 24-h incubation experiment performed in duplicate. The IC_50_ value is the concentration of a tested substance that causes a 50% decrease in cell viability relative to the negative control, which is considered 100% (*N*=2).

**Figure S4. MTT assay results:** Dose-response curves obtained for **MM134**, **-136**, **-137**, and -**139** compounds in DLD-1 colorectal cancer cells in a 24-h incubation experiment performed in duplicate. The IC_50_ value is the concentration of a tested substance that causes a 50% decrease in cell viability relative to the negative control, which is considered 100% (*N* =2).

**Figure S5. MTT assay results:** Dose-response curves obtained for **MM134**, **-136**, **-137**, and -**139** compounds in CCD-841CoN large intestine normal cells in a 24-h incubation experiment performed in duplicate. The IC_50_ value is the concentration of a tested substance that causes a 50% decrease in cell viability relative to the negative control, which is considered 100%.

**Figure S6.** Images of comets obtained in an alkaline comet assay following 24-h treatment of DLD-1 cells with **MM134**, **-136**, **-137,** and **-139** compounds used in respective consensus IC_50_ concentrations and bleomycin used in 10 μM concentration (arbitrary magnification).

**Figure S7.** Images of comets obtained in an alkaline comet assay following 24-h treatment of HCT-116 cells with **MM134**, **-136**, **-137,** and **-139** compounds used in respective consensus IC_50_ concentrations and bleomycin used in 10 μM concentration (arbitrary magnification).

**Figure S8.** Images of comets obtained in an alkaline comet assay following 24-h treatment of HT-29 cells with **MM134**, **-136**, **-137,** and **-139** compounds used in respective consensus IC_50_ concentrations and bleomycin used in 10 μM concentration (arbitrary magnification).

**Figure S9.** Images of comets obtained in an neutral comet assay following 24-h treatment of HCT-116 cells with **MM134**, **-136**, **-137,** and **-139** compounds used in respective consensus IC_50_ concentrations and bleomycin used in 10 μM concentration (arbitrary magnification).

**Table S3.** Pro-necrotic properties of pyrazolo[4,3-*e*]tetrazolo[1,5-*b*][1,2,4]triazine sulphonamides (**MM** compounds) in HCT-116 and HT-29 cells using dual acridine orange/ethidium bromide (AO/EB), Hoechst 33342/propidium ioide (HE/PI) fluorescent staining and flow cytometry analysis with annexin V-FITC/PI staining. Result were presented as a mean percentage [%] ± SD of necrotic cells in cells treated with IC_50_ concentrations of **MM134**, **-136**, **-137** and **-139** compounds. Dunnett’s test was used to represent statistically significant changes (*p* < 0.05) in the mean % of necrotic cells in treated samples compared with a negative control. Statistically significant changes in necrotic cell fraction were indicated as bolds.

| **Method** | **Cell line** | **Incubation time** | **Control** | **MM134** | **MM136** | **MM137** | **MM139** |
| --- | --- | --- | --- | --- | --- | --- | --- |
| **Acridine orange/ethidium bromide (AO/EB) staining** | HCT-116 | 24-h | 9 ± 0.5 | 11 ± 1 | 3.3 ± 1.5 | 8.3 ± 0.6 | 10.3 ± 0.6 |
|  |  | 48-h | 8 ± 2.6 | 10 ± 2 | 2.7 ± 2 | 7 ± 2.6 | 10.7 ± 1.2 |
|  | HT-29 | 24-h | 14 ± 1.7 | 17.3 ± 2 | 10.1 ± 0.6 | 9.7 ± 0.6 | 12.3 ± 2.5 |
|  |  | 48-h | 5.3 ± 0.6 | 10.7 ± 3 | **14.6 ± 2.4** | **20 ± 2** | 10.3 ± 2.5 |
| **Hoechst 33342/propidium ioide (HE/PI) staining** | HCT-116 | 24-h | 15.7 ± 2 | 16 ± 1 | 14.3 ± 1.5 | 14.7 ± 3.8 | 13.7 ± 1.5 |
|  |  | 48-h | 5.7 ± 1.2 | 9.5 ± 0.5 | 6.3 ± 1.5 | 8.3 ± 1.5 | 8 ± 2 |
|  | HT-29 | 24-h | 16.7 ± 1.5 | 21 ± 1 | **25.3 ± 1.5** | **19.3 ± 1.2** | 21 ± 1 |
|  |  | 48-h | 12.2 ± 1.8 | 18.8 ± 1.6 | 13.5 ± 1.5 | 14.2 ± 2.3 | 15.7 ± 3 |
| **Flow cytometry analysis with annexin V-FITC/PI staining** | HCT-116 | 24-h | 1.2 ± 0.6 | 1.6 ± 0.4 | **8.5 ± 1.7** | 1.8 ± 0.5 | 2 ± 0.7 |
|  |  | 48-h | 7 ± 2.8 | 11 ± 1.4 | 3 ± 2.8 | 7.5 ± 3.5 | 11 ± 1.4 |
|  | HT-29 | 24-h | 0.7 ± 0.2 | 0.5 ± 0.3 | 0.5 ± 0.3 | 0.5 ± 0.2 | 0.3 ± 0.1 |
|  |  | 48-h | 5.4 ± 0.8 | 11 ± 4.2 | 11.6 ± 7.2 | 10 | 10.5 ± 3.5 |

**Table S4.** Pro-apoptotic properties of pyrazolo[4,3-*e*]tetrazolo[1,5-*b*][1,2,4]triazine sulphonamides (**MM** compounds) in HCT-116 and HT-29 cells using dual acridine orange/ethidium bromide (AO/EB), Hoechst 33342/propidium ioide (HE/PI) fluorescent staining and flow cytometry analysis with annexin V-FITC/PI staining. Result were presented as a mean percentage [%] ± SD of necrotic cells in cells treated with 0.5 μM concentrations of **MM134**, **-136**, **-137,** and **-139** compounds. Dunnett’s test was used to represent statistically significant changes (*p* < 0.05) in the mean % of necrotic cells in treated samples compared with a negative control. Statistically significant changes in necrotic cell fraction were indicated as bolds.

| **Apoptosis detection method** | **Cell line** | **Incubation time** | **Control** | **MM134** | **MM136** | **MM137** | **MM139** |
| --- | --- | --- | --- | --- | --- | --- | --- |
| **Acridine orange/ethidium bromide (AO/EB) staining** | HCT-116 | 24-h | 0.66 ± 0.3 | **38.5 ± 1.3** | **43.3 ± 2** | **45.9 ± 2.7** | 19.33 ± 5.5 |
|  |  | 48-h | 6.33 ± 1.53 | **65.3 ± 2.5** | **77.5 ± 2.8** | **85 ± 3** | **47.3 ± 2** |
|  | HT-29 | 24-h | 1.2 ± 0.8 | **32.3 ± 1.5** | **24.7 ± 2** | **21.7 ± 3.5** | **13.3 ± 1.6** |
|  |  | 48-h | 9.13 ± 2.2 | 53.8 ± 9.8 | **69.3 ± 9.9** | **47.8 ± 3.8** | 36.7 ± 9 |
| **Hoechst 33342/propidium ioide (HE/PI) staining** | HCT-116 | 24-h | 1.6 ± 0.4 | **45.3 ± 3** | **49.7 ± 2.5** | **53.7 ± 1.5** | **16.7 ± 2** |
|  |  | 48-h | 10.8 ± 1 | **65 ± 3** | **63 ± 1.7** | **86 ± 1.7** | **42.3 ± 2.5** |
|  | HT-29 | 24-h | 3.5 ± 1.3 | **30 ± 2** | **22.7 ± 2.5** | **31.7 ± 2.5** | **15.3 ± 3** |
|  |  | 48-h | 13.7 ± 1.5 | **50.2 ± 2.2** | **52.5 ± 2.8** | **61.7 ± 3.5** | **36.7 ± 2** |
| **Flow cytometry analysis with annexin V-FITC/PI staining** | HCT-116 | 24-h | 5.2 ± 0.3 | **12.2 ± 1.3** | **21.3 ± 4** | **32 ± 4.6** | 8 ± 2.9 |
|  |  | 48-h | 5.5 ± 0.7 | **64 ± 1.4** | **76.2 ± 2.5** | **83.5 ± 2.12** | 46.5 ± 2.12 |
|  | HT-29 | 24-h | 15.8 ± 0.1 | 30.4 ± 7.7 | **32.2 ± 5.2** | **36 ± 4** | **34.6 ± 4** |
|  |  | 48-h | 9.2 ± 2.5 | **37 ± 4.6** | **33.2** ± 5.5 | **39.5** ± 5.2 | 23 ± 4.6 |

**Table S5.** Pro-necrotic properties of pyrazolo[4,3-*e*]tetrazolo[1,5-*b*][1,2,4]triazine sulphonamides (**MM** compounds) in HCT-116 and HT-29 cells using dual acridine orange/ethidium bromide (AO/EB), Hoechst 33342/propidium ioide (HE/PI) fluorescent staining and flow cytometry analysis with annexin V-FITC/PI staining. Result were presented as a mean percentage [%] ± SD of necrotic cells in cells treated with 0.5 μM concentrations of **MM134**, **-136**, **-137,** and **-139** compounds. Dunnett’s test was used to represent statistically significant changes (*p* < 0.05) in the mean % of necrotic cells in treated samples compared with a negative control. Statistically significant changes in necrotic cell fraction were indicated as bolds.

| **Method** | **Cell line** | **Incubation time** | **Control** | **MM134** | **MM136** | **MM137** | **MM139** |
| --- | --- | --- | --- | --- | --- | --- | --- |
| **Acridine orange/ethidium bromide (AO/EB) staining** | HCT-116 | 24-h | 9 ± 0.5 | **26.3 ± 3.2** | **23 ± 3** | **35.7 ± 3.5** | **18.7 ± 1.5** |
|  |  | 48-h | 8 ± 2.6 | 21 ± 1.7 | 6.4 ± 0.6 | 10.7 ± 1.1 | 19.3 ± 5.1 |
|  | HT-29 | 24-h | 14 ± 1.7 | 14 ± 1.7 | 27 ± 2 | 26 ± 2 | 10.3 ± 0.6 |
|  |  | 48-h | 5.3 ± 0.6 | **20.3 ± 2.4** | 15.3 ± 5 | **23.9 ± 1.7** | **19.3 ± 2** |
| **Hoechst 33342/propidium ioide (HE/PI) staining** | HCT-116 | 24-h | 15.7 ± 2 | 22 ± 2 | 21 ± 1 | **22.7 ± 2.5** | 19.7 ± 2.5 |
|  |  | 48-h | 5.7 ± 1.2 | 16.3 ± 1.5 | 10.3 ± 1.5 | 11 ± 1 | 10 ± 1 |
|  | HT-29 | 24-h | 16.7 ± 1.5 | **31 ± 3.6** | **29 ± 1** | **23 ± 1** | **27.5 ± 0.9** |
|  |  | 48-h | 12.2 ± 1.8 | 22.5 ± 2.5 | 16.8 ± 1.3 | 18.2 ± 0.3 | 19.7 ± 2.5 |
| **Flow cytometry analysis with annexin V-FITC/PI staining** | HCT-116 | 24-h | 1.2 ± 0.6 | 1.9 ± 0.6 | 4.6 ± 2.1 | 7.4 ± 2 | 2.9 ± 3.3 |
|  |  | 48-h | 7 ± 2.8 | **21.5 ± 2** | 6.6 ± 0.6 | 11 ± 1.4 | **20 ± 7** |
|  | HT-29 | 24-h | 0.7 ± 0.2 | 1.6 ± 1.3 | 4.3 ± 3.4 | 2.7 ± 2.4 | 2.5 ± 2.3 |
|  |  | 48-h | 5.4 ± 0.8 | **24.4 ± 2** | 9.5 ± 2.1 | **23.4 ± 2** | 19 ± 2.8 |

**Table S6.** Genetic and molecular differences between DLD-1, HCT-116, and HT-29 cells. .

| **Cell line** | **DLD-1** | **HCT-116** | **HT-29** |
| --- | --- | --- | --- |
| **Type** | Adenocarcinoma | Adenocarcinoma | Adenocarcinoma |
| **Tumorigenic** | Yes | Yes | Yes |
| **Source/note** | Adult Male | Adult Male | Adult Female |
| **Ploidy** | Pseudo-diploid | Near-diploid | Hyper-triploid |
| **TP53** | Mutated (p.S241F) | WT | Mutated (p.R273H) |
| **APC** | Mutated (traucated) | WT | Mutated (traucated) |
| **KRAS** | Mutated (p.G13D) | Mutated (p.G13D) | WT |
| **BRAF** | WT | WT | Mutated |
| **PIK3CA** | Mutated (p.E545K; p.D549N) | Mutated (p.H1047R) | WT |
| **PTEN** | WT | WT | WT |
| **CMS** | CMS1 (immune) | CMS4 (mesenchymal) | CMS3 (metabolic) |
| **MSI** | MSI | MSI | MSS |
| **CIMP** | Yes | Yes | Yes |
| **POLE** | WT | WT | WT |
| **Colon-like/undifferentiated** | Undifferentiated | Undifferentiated | Colon-like |

APC – adenomatous polyposis coli; BRAF – B-rapidly accelerated fibrosarcoma; CIMP – CpG island methylator phenotype; CMS - consensus molecular subtypes; KRAS – Kirsten rat sarcoma viral oncogene homolog; MSI - microsatellite instability; MSS - microsatellite stability; PIK3CA – phosphatidylinositol-4,5-bisphosphate 3-kinase catalytic subunit alpha; POLE – DNA polymerase epsilon, catalytic subunit; PTEN – phosphatase and tensin homolog; TP53 – cellular tumor antigen p53; WT – wild type
